# Pan-cancer and multi-omics analyses revealed the diagnostic and prognostic value of BAZ2A in liver cancer

**DOI:** 10.1038/s41598-024-56073-7

**Published:** 2024-03-04

**Authors:** Yan Liu, Junli Wang, Jimin Guo, Qianyi Zhang, Shuqing Wang, Fen Hu, Jinghua Wu, Yating Zhao, Jinghua Zhang, Yuan Yu, Yufeng Li, Xiaojun Zhang

**Affiliations:** 1 College of Life Science, North China University of Science and Technology, Tangshan, 063210 China; 2 Hebei Key Laboratory of Molecular Oncology, Tangshan, 063001 China; 3 School of Public Health, North China University of Science and Technology, Tangshan, 063210 China; 4 Hospital of North China University of Science and Technology, Tangshan, 063210 China; 5 Department of Clinical Laboratory, North China University of Science and Technology Affiliated Tangshan Maternal and Child Health Hospital, Tangshan, 063000 China; 6 Department of Breast Center, North China University of Science and Technology Affiliated Hospital, Tangshan, 063210 China; 7 The Cancer Institute, Tangshan People’s Hospital, Tangshan, 063001 China; 8 Department of Oncology, People’s Hospital of Zunhua, Tangshan, 064200 China

**Keywords:** BAZ2A, Enrichment analysis, Pan-cancer, Prognosis, LIHC, Cancer, Cell biology, Computational biology and bioinformatics, Molecular biology

## Abstract

BAZ2A, an epigenetic regulatory factor that affects ribosomal RNA transcription, has been shown to be highly expressed in several cancers and promotes tumor cell migration. This study explored the expression and mechanism of BAZ2A in tumorigenesis at the pan-cancer level. The Cancer Genome Atlas, Gene Expression Omnibus databases and TIMER2.0, cBioPortal and other tools were used to analyze the level of expression of BAZ2A in various tumor tissues and to examine the relationship between *BAZ2A* and survival, prognosis, mutation and immune invasion. In vitro experiments were performed to assess the function of *BAZ2A* in cancer cells. Using combined transcriptome and proteome analysis, we examined the possible mechanism of BAZ2A in tumors. BAZ2A exhibited high expression levels in multiple tumor tissues and displayed a significant association with cancer patient prognosis. The main type of *BAZ2A* genetic variation in cancer is gene mutation. Downregulation of *BAZ2A* inhibited proliferation, migration, and invasion and promoted apoptosis in LM6 liver cancer cell. The mechanism of BAZ2A in cancer development may involve lipid metabolism. These results help expand our understanding of BAZ2A in tumorigenesis and development and suggest BAZ2A may serve as a prognostic and diagnostic factor in several cancers.

## Introduction

Cancer poses a global public health challenge, with a notable surge in both the occurrence and fatality rates of this disease^1,2^. Remarkable progress has been achieved in the advancement of cancer treatment strategies, but the efficacy of treatment and prognosis of cancer patients are still not ideal because of drug resistance and the potential adverse effects of drugs^3^. Hence, further investigation of the pathogenesis of cancer is critical to develop new therapies to improve patient prognosis^4^.

Bromodomain adjacent to zinc finger domain protein 2 (*BAZ2A*, also known as TIP5 or WALp3) is a member of the BAZ family. BAZ2A is an epigenetic regulator that affects ribosomal RNA (rRNA) transcription^5^ and binds to the DNA-dependent adenosine triphosphatase SNF2H to form the nucleolar remodeling complex, which is responsible for the formation of rDNA heterochromatin^6,7^. BAZ2A is composed of 1,908 amino acid residues; the PHD-BRD tandem domain located at the C-terminal is the most widely studied and mediates rDNA silencing^8,9^.

BAZ2A promotes the invasion of tumor cells and has cancer promoting activities^10,11^. BAZ2A was shown to promote epigenetic changes in aggressive cancers. BAZ2A is highly expressed in prostate cancer (PCa), and its overexpression predicts cancer occurrence^12^. *BAZ2A* is a target gene of multiple microRNAs that are involved in the pathogenesis and metastasis of pancreatic adenocarcinoma, bladder carcinoma and prostate adenocarcinoma^11^.

According to a study, the activation of the β-catenin/transcription pathway is implicated in the promotion of hepatocellular carcinoma (HCC) by BAZ2A^10^. While there are many studies on BAZ2A, most of them are focused on its role in a specific cancer.

Protein phosphorylation is the most frequently occurring post-translational modification^13,14^. The level of phosphorylation modification is also related to the progression of many cancers^15^. DNA methylation is an important epigenetic modification that determines the expression of many genes^16^. Abnormal DNA methylation is thought to be associated with tumorigenesis, with genome-wide DNA hypomethylation observed in cancer tissues compared to non-tumor tissues^17^.

This study aimed to investigate the pan-cancer level expression of BAZ2A and evaluate its prognostic capability, with the aim of identifying cancers in which *BAZ2A* may function as a diagnostic and prognostic marker.

## Results

### Expression of BAZ2A in pan-cancer

**Figure 1:**
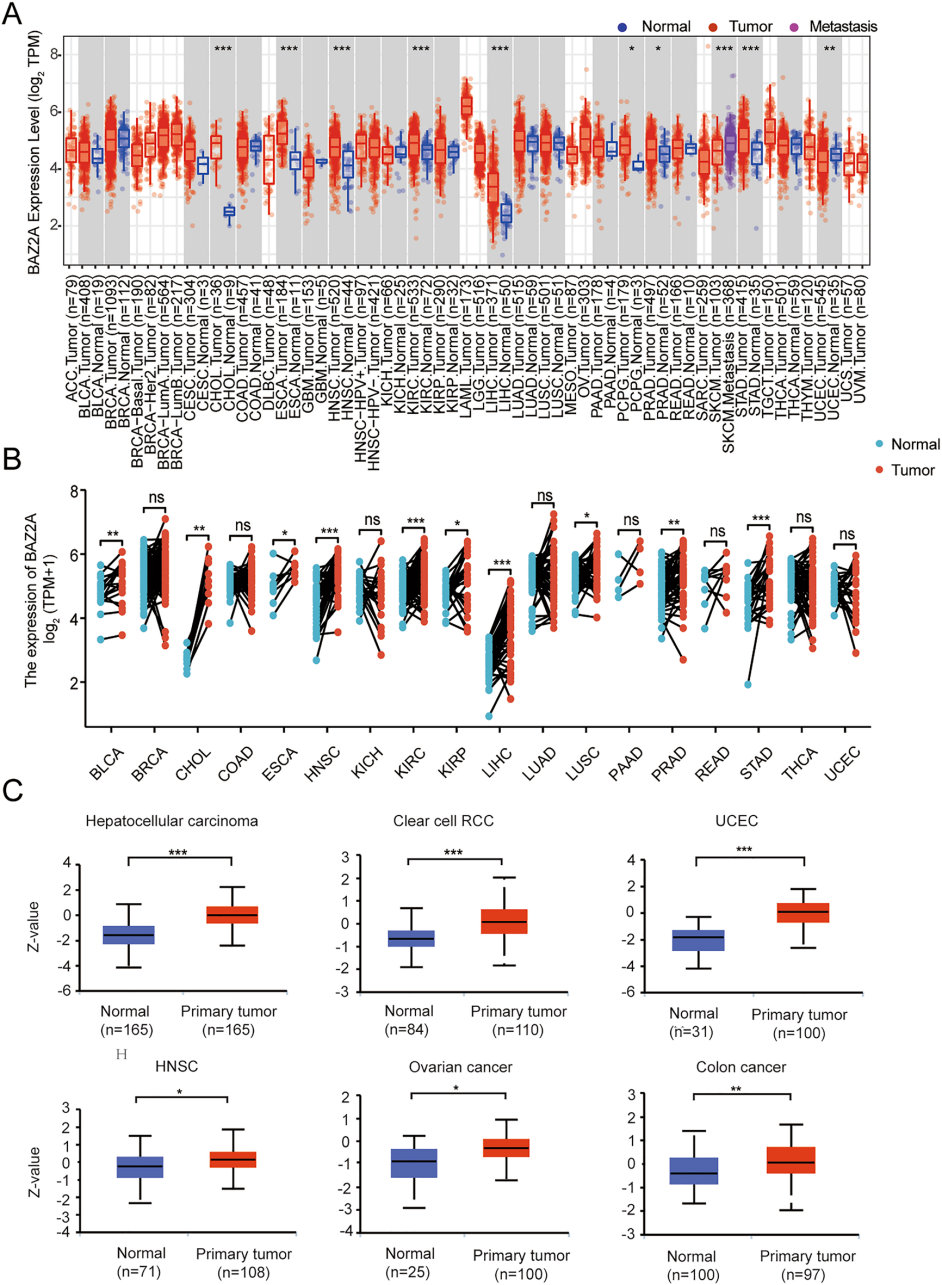
The expression patterns of BAZ2A in various types of cancers. (**A**) We conducted an analysis of BAZ2A expression across multiple cancer types using the TIMER database. *P < 0.05; **P < 0.01; ***P < 0.001. *P* < 0.05 is considered as a statistical difference. (**B**) Analysis of *BAZ2A* expression in pan-carcinoma using paired paracancerous/tumor samples in TCGA database. *P < 0.05, **P < 0.005, ***P < 0.001. The abbreviation “ns” indicates non-significant results. (**C**) Analysis of BAZ2A protein expression levels in the above six cancers using UALCAN. *P < 0.05, **P < 0.005, ***P < 0.001.

First, in Fig. 1A, we used the TCGA database to analyze the expression level of BAZ2A in cancer tissues and adjacent normal tissues, and analyzed and tested the unmatched samples from 33 cancer species. In Fig. 1A, we also showed the sample size n for each group. The results showed that the expression level of BAZ2A in cancer tissues was higher than that in normal tissues in most cancer species. These include cholangiocarcinoma (CHOL), esophageal cancer (ESCA), head and neck squamous cell carcinoma (HNSC), kidney renal clear cell carcinoma (KIRC), Kidney renal papillary cell carcinoma (KIRP), liver hepatocellular carcinoma (LIHC), Pheochromocytoma and Paraganglioma (PCPG), prostate adenocarcinoma (PRAD), Skin Cutaneous Melanoma (SKCM), stomach adenocarcinoma (STAD) (Fig. 1A, Table 1). By contrast, in Uterine Corpus Endometrial Carcinoma (UCEC), BAZ2A showed abnormally low expression levels, which may be related to the diversity of BAZ2A expression in human tumors or the tissue specificity of endometrial carcinoma^18^.

**Table 1:** Screening process of cancer species.

		ACC	BLCA	BRCA	CESC	CHOL	COAD	DLBC	ESCA	GBM	HNSC	KICH	KIRC	KIRP	LAML	LGG	LIHC	LUAD	LUSC	MESO	OV	PCPG	PRAD	PEAD	SARC	SKCM	STAD	TGCT	THCA	THYM	UCEC	USC	UVM	PAAD	Colon cancer
mRNA expression	F1A unpaired sample	ns	ns	ns	ns	^⭐⭐⭐^	ns	ns	^⭐⭐⭐^	ns	^⭐⭐⭐^	ns	^⭐⭐⭐^	ns	ns	ns	^⭐⭐⭐^	ns	ns	ns	ns	^⭐^	^⭐^	ns	ns	ns	^⭐⭐⭐^	ns	ns	ns	ns	^⭐⭐^	ns	//	//
	F1B Paired sample	//	^⭐⭐^	ns	//	//	ns	//	^⭐^	//	^⭐⭐⭐^	ns	^⭐⭐⭐^	^⭐^	//	//	^⭐⭐⭐^	ns	^⭐^	//	//	//	^⭐⭐^	ns	//	//	^⭐⭐⭐^	//	ns	//	ns	//	//	ns	//
Protein expression	F1C	//	//	//	//	//	//	//	//	//	//	//	^⭐⭐⭐^	//	//	//	^⭐⭐⭐^	//	//	//	^⭐^	//	//	//	//	//	//	//	//	//	^⭐⭐⭐^	//	//	//	^⭐⭐^
OS (*P* value)	F2A	//	//	//	//	//	//	//	//	//	//	//	0.031	0.0088	//	//	0.00027	//	//	//	//	0.0053	//	//	//	//	//	//	//	//	0.028	//	//	//	//
DSS (*P* value)	F2B	//	//	//	//	//	//	//	//	//	//	//		0.02	//	//	0.0077	//	//	//	//		//	//	//	//	//	//	//	//		//	//	//	//
ROC curve (AUC)	F2C	//	//	//	//	//	//	//	//	//	//	//	0.642	0.522	//	//	0.827	//	//	//	//	0.741	//	//	//	//	//	//	//	//	0.61	//	//	//	//
Methylation (*P* value)	F3A up	//	//	//	//	//	//	//	//	//	//	//	0.042	0.001	//	//	0.001	//	//	//	//	0.015	//	//	//	//	//	//	//	//	0.257	//	//	//	//
	F3A down	//	//	//	//	//	//	//	//	//	//	//	0.989	0.001	//	//	0.002	//	//	//	//	0.226	//	//	//	//	//	//	//	//	0.001	//	//	//	//
Immunoinfiltration (*P* value)	F4B purity	//	//	//	//	//	//	//	//	//	//	//	5.52E−01	5.87 E−01	//	//	2.14 E−01	//	//	//	//	4.11 E−03	//	//	//	//	//	//	//	//	7.62 E−01	//	//	//	//
	F4B B cell	//	//	//	//	//	//	//	//	//	//	//	8.66 E−07	4.02 E−01	//	//	2.24 E−15	//	//	//	//	6.25 E−02	//	//	//	//	//	//	//	//	1.16 E−01	//	//	//	//
	F4B CD8 T cell	//	//	//	//	//	//	//	//	//	//	//	7.15 E−08	5.79 E−01	//	//	3.39 E−10	//	//	//	//	2.46 E−01	//	//	//	//	//	//	//	//	1.02 E−03	//	//	//	//
	F4B CD4 T cell	//	//	//	//	//	//	//	//	//	//	//	1.26 E−33	8.75 E−03	//	//	8.00 E−24	//	//	//	//	1.18 E−01	//	//	//	//	//	//	//	//	2.95 E−02	//	//	//	//
	F4B Macrophage	//	//	//	//	//	//	//	//	//	//	//	2.32 E−13	3.90 E−01	//	//	5.52 E−27	//	//	//	//	1.54 E−03	//	//	//	//	//	//	//	//	2.19 E−02	//	//	//	//
	F4B Neutrophil	//	//	//	//	//	//	//	//	//	//	//	1.76 E−29	1.87 E−05	//	//	1.08 E−28	//	//	//	//	8.59 E−03	//	//	//	//	//	//	//	//	3.67 E−10	//	//	//	//
	F4B Dendritic cell	//	//	//	//	//	//	//	//	//	//	//	1.01 E−13	9.01 E−04	//	//	9.71 E−22	//	//	//	//	6.94 E−01	//	//	//	//	//	//	//	//	3.73 E−03	//	//	//	//
Coexpressed gene	F5D	//	//	//	//	//	//	//	//	//	//	//	^⭐⭐⭐^	^⭐⭐⭐^	//	//	^⭐⭐⭐^	//	//	//	//	^⭐⭐⭐^	//	//	//	//	//	//	//	//	^⭐⭐⭐^	//	//	//	//

^⭐^*P* < 0.05; ^⭐⭐^*P* < 0.01; ^⭐⭐⭐^*P* < 0.001.

*P* < 0.05 is considered as a statistical difference; The abbreviation “ns” indicates non-significant results.

//: There is no sample data for this cancer in the corresponding database/this cancer type is not used.

Secondly, in Fig. 1B, paired samples of 18 cancer species in the TCGA database were used for further analysis and testing. The results showed that In Bladder Urothelial Carcinoma (BLCA), CHOL, ESCA, HNSC, KIRC, KIRP, LIHC, Lung squamous cell carcinoma (LUSC), PRAD, In STAD, the expression level of BAZ2A was significantly increased in cancer tissues (Fig. 1B, Table 1).Finally, combined with Fig. 1A,B, we found that BAZ2A showed high expression levels in multiple cancer species in both paired and unpaired samples.

CPTAC analysis showed that BAZ2A protein expression was increased in diverse types of cancer (Fig. 1C). To determine the association between BAZ2A expression levels and tumor pathological stage, we evaluated BAZ2A expression in patients using CPTAC analysis and found that upregulation of BAZ2A protein expression correlated with pathological stage of clear cell carcinoma, UCEC, ovarian cancer, and colon cancer (Supplementary Fig. 1A). Together, these findings demonstrate that Elevated BAZ2A expression was observed in cancerous tissues and exhibited a correlation with pathological stage.

### BAZ2A phosphorylation in pan-cancer

Using the CPTAC dataset, we analyzed the levels of BAZ2A phosphorylation in four cancer types (LIHC, KIRC, OV, and HNSC). The S1395 site of BAZ2A showed higher phosphorylation levels in LIHC and KIRC tumor tissues in comparison to normal tissues, but lower phosphorylation levels in OV tumor tissues (Supplementary Fig. 1B). S1768 phosphorylation levels were also higher in LIHC, OV, HNSC tumor tissues compared with normal tissues. Our results also revealed several sites with increased phosphorylation in HNSC tumor tissues, including S115, S494, S1768, Y1775, and S1776. These findings imply that alterations in the post-translational modifications of BAZ2A might contribute to the process of tumorigenesis.

### The association between BAZ2A expression and tumor diagnosis and prognosis

**Figure 2:**
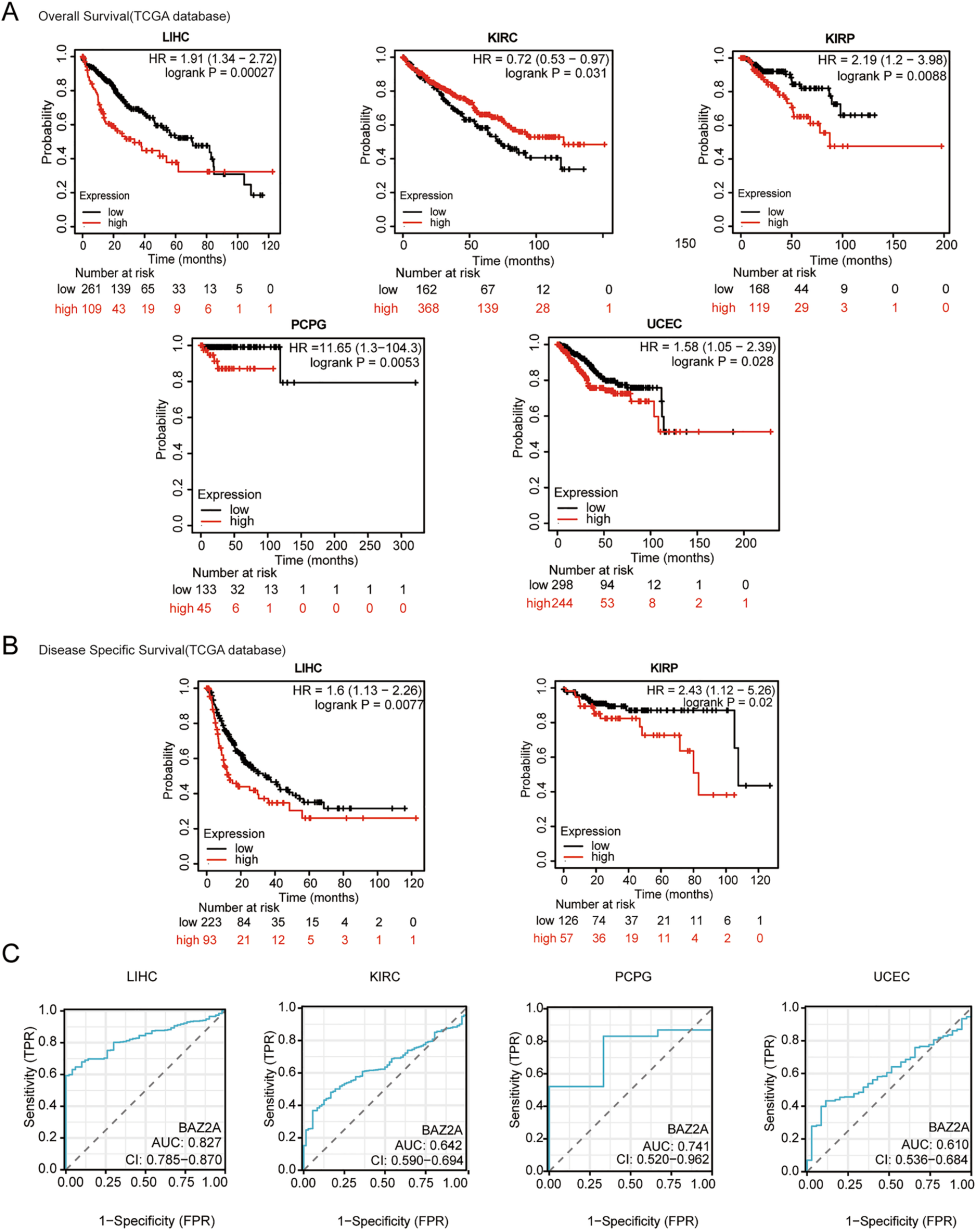
Cancer prognostic and diagnostic analyses based on *BAZ2A* expression. (**A**) The relationship between BAZ2A mRNA expression and overall survival in TCGA and GEO datasets. *P* < 0.05 is considered as a statistical difference. (**B**) The relationship between *BAZ2A* mRNA expression and disease-free survival was evaluated using the TCGA dataset. *P* < 0.05 is considered as a statistical difference. (**C**) ROC analysis of cancer diagnosis based on BAZ2A mRNA expression levels.

Subsequently, we used the TCGA dataset to examine the association between BAZ2A expression and patient outcomes in 21 different cancers. Figure 2A,B showed statistically significant cancer types. The survival analysis indicated that increased expression of BAZ2A was linked to a poor prognosis in patients with LIHC, KIRP, pheochromocytoma and paraganglioma (PCPG), and endometrial cancer (UCEC), while reduced expression of *BAZ2A* was linked to an adverse prognosis in KIRC (Fig. 2A,B).

ROC curves were employed to assess the diagnostic utility of BAZ2A across multiple cancer types. *BAZ2A* showed some diagnostic accuracy for LIHC (0.846), KIRC (0.642), PCPG (0.741), and UCEC (0.61) (Fig. 2C). These results showed that *BAZ2A* expression influenced prognosis in several cancers and that *BAZ2A* gene expression may serve as a diagnostic marker in some cancers.

### Genetic alterations are correlated with DNA methylation of the BAZ2A promoter

**Figure 3:**
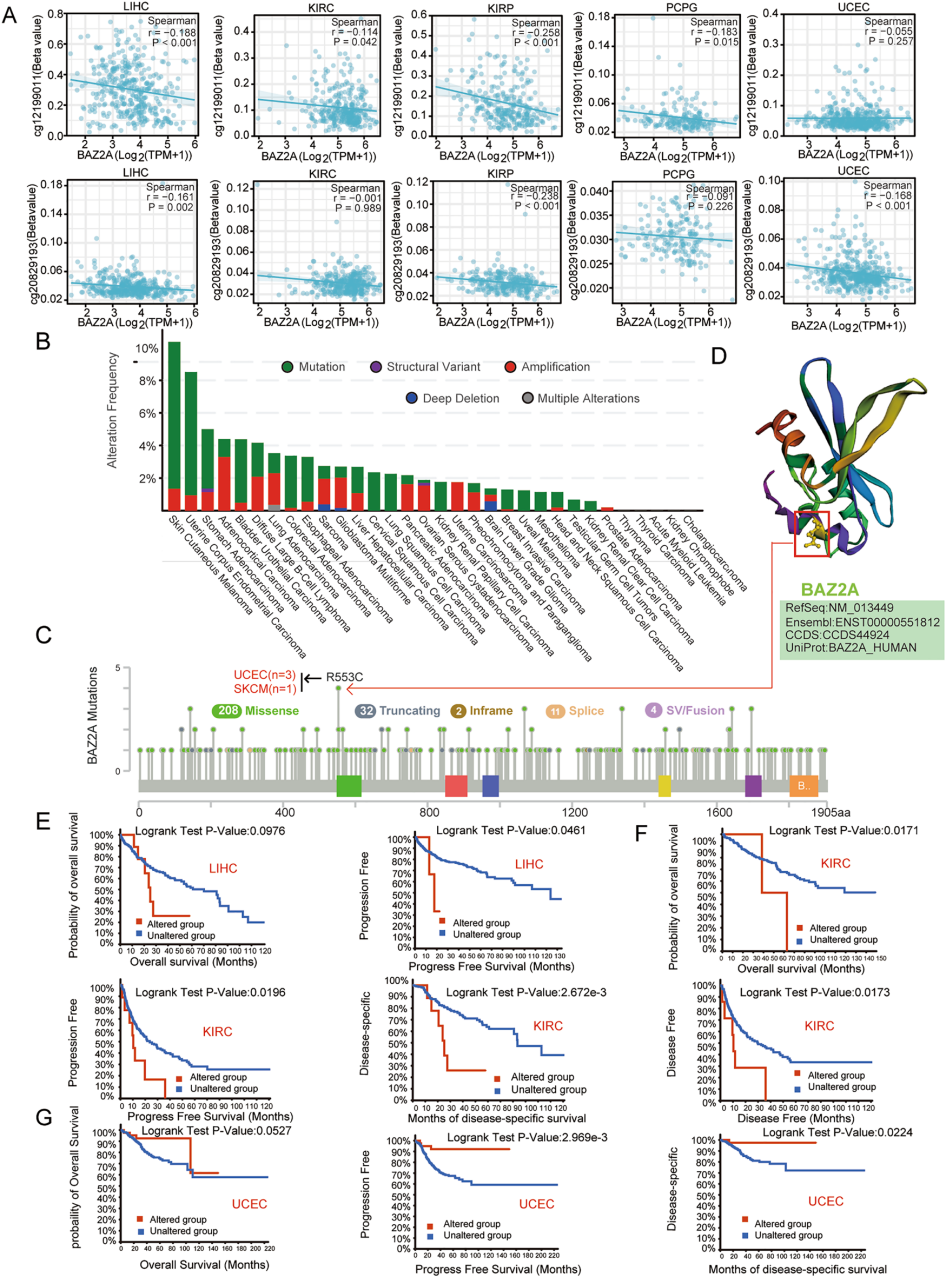
DNA methylation analysis and characterization of BAZ2A genetic variations in cancer. (**A**) BAZ2A promoter methylation levels correlated with BAZ2A mRNA levels in LIHC, KIRC, KIRP, PCPG, and UCEC. *P* < 0.05 is considered as a statistical difference. (**B**) The cBioPortal database was used to examine BAZ2A mutations in pan-cancer, and the frequency of different types of BAZ2A mutations in each cancer is shown. (**C**) The mutation sites and mutation types of BAZ2A gene variations in cancer and the corresponding number of cases in TCGA. (**D**) The three-dimensional structure of BAZ2A and the most frequently mutated site (R553C) in cancer. (**E**–**G**) We employed the cBioPortal database to study the effects of BAZ2A mutation status on various survival outcomes, including overall survival, disease-free survival, progression-free survival, and disease-specific survival in LIHC (**E**), KIRC (**F**), and UCEC (**G**). *P* < 0.05 is considered as a statistical difference.

We used two probes (cg12199011, cg20829193) to analyze TCGA RNAseq and Methylation450 data to detect DNA methylation levels of the *BAZ2A* gene promoter. The findings demonstrated that *BAZ2A* expression was inversely linked to promoter methylation in LIHC, KIRC, KIRP, PCPG, and UCEC (Fig. 3A).

Analysis of the gene alteration frequency revealed a variety of genetic variations in the *BAZ2A* gene in cancer, such as mutations, structural variations, amplification, and deletions, among which mutation was the most frequently detected variation, including missense mutation, truncating mutation, inframe mutation, splice mutation, fusion mutation (Fig. 3B,C). Cutaneous melanoma (SKCM) exhibited the highest frequency of BAZ2A gene alteration (> 10%) (Fig. 3B). The sites and types of *BAZ2A* gene alterations are shown in Fig. 3C. Missense mutation were the major categories of genetic alterations; the R553C mutation was detected in three UCEC samples and one SKCM sample (Fig. 3C,D).

Survival analysis indicated that patients with *BAZ2A* gene mutations had a poorer prognosis in terms of OS, PFS, DFS, DSS, and DSS in LIHC and KIRC than patients without *BAZ2A* gene mutation (Fig. 3E,F). In UCEC, the OS, PFS, DSS of patients with *BAZ2A* gene mutations were better than those without *BAZ2A* gene mutation (Fig. 3G).

### Association of BAZ2A gene expression and immune infiltration

**Figure 4:**
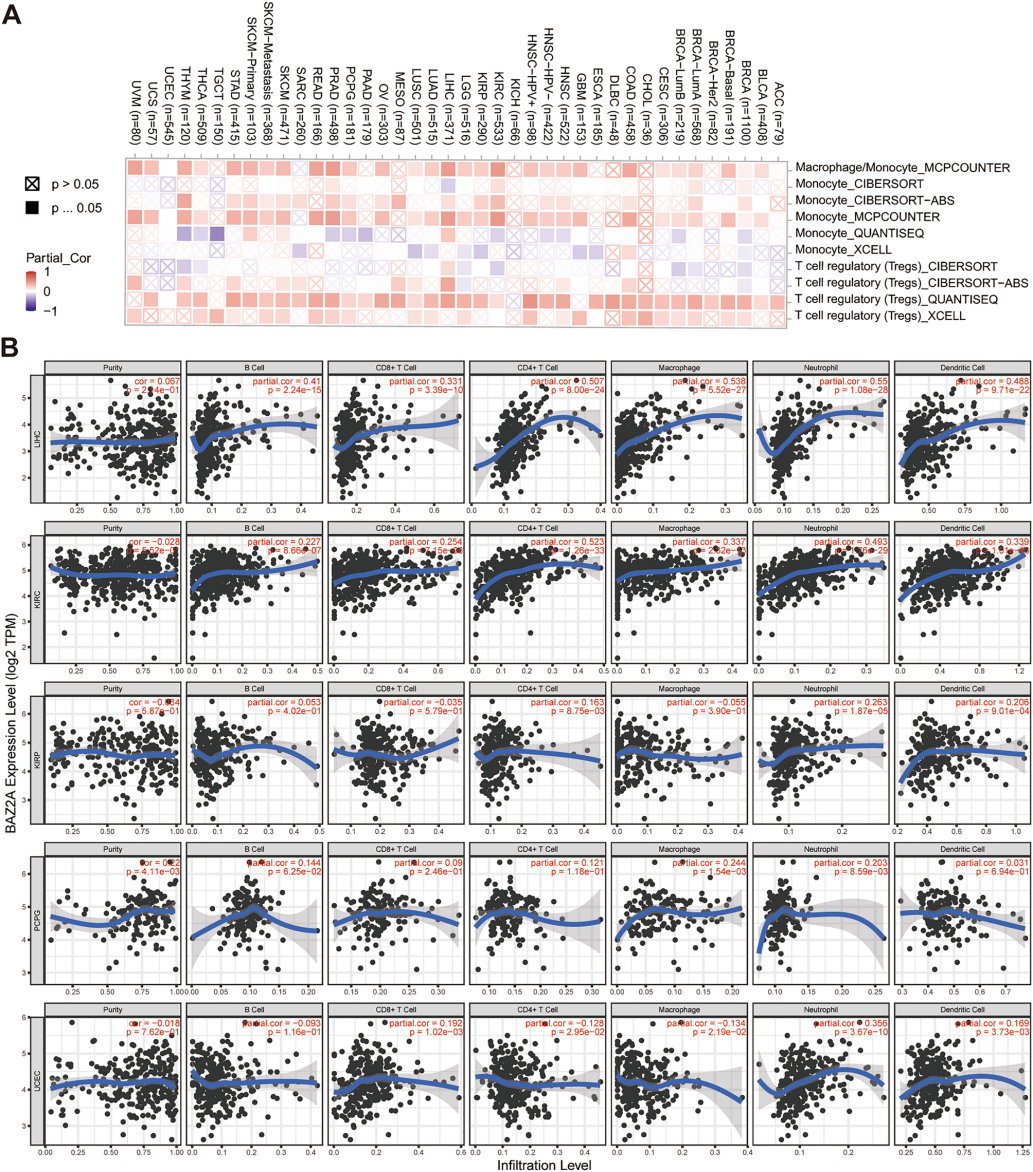
Correlation analysis of BAZ2A expression and immune cell infiltration in cancer. (**A**) Correlation between BAZ2A expression and infiltration of T regulatory cells and macrophages using the TIMER2.0 database. *P* < 0.05 is considered as a statistical difference. (**B**) Correlation of BAZ2A expression with levels of the infiltration of immune cells in LIHC, KIRC, KIRP, PCPG, and UCEC. *P* < 0.05 is considered as a statistical difference.

Next, we used a timer platform to assess the relationship between BAZ2A gene expression and the degree of immune cell infiltration in 31 cancers. In most cancers, including LIHC, KIRC, KIRP, PCPG, and HNSC (including HNSC+HPV−, HNSC+HPV+), we observed a positive correlation between BAZ2A mRNA expression and the levels of Tregs and macrophage cell infiltration (Fig. 4A). The scatterplots generated by the TIMER also demonstrated that *BAZ2A* expression exhibited a association with the level of invasion of immune cells in different tumors (Fig. 4B). Taken together, BAZ2A gene expression is positively correlated with immune cell infiltration in multiple cancer types.

### Enrichment analysis of proteins and genes associated with BAZ2A

**Figure 5:**
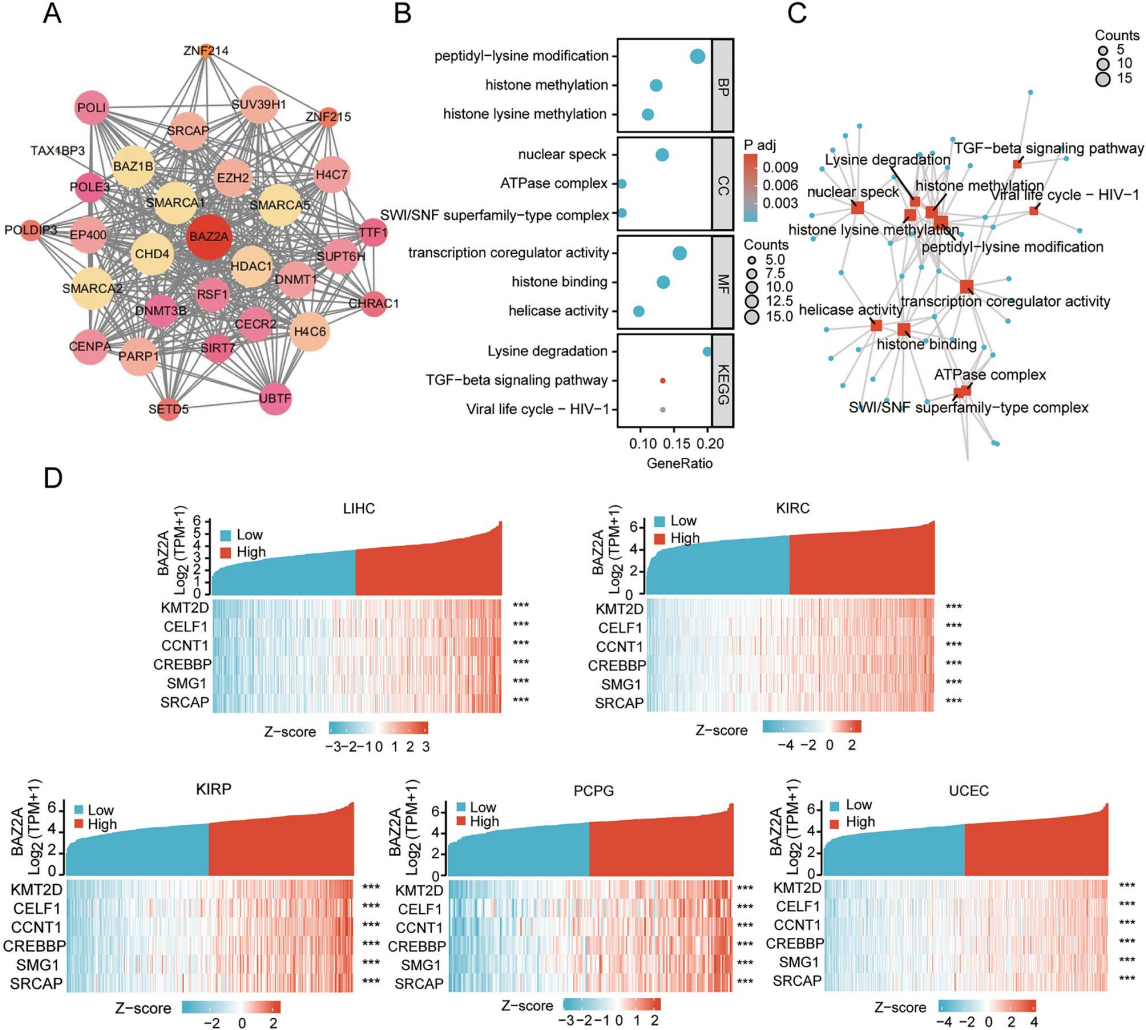
Enrichment analysis of BAZ2A-associated proteins and genes. (**A**) BAZ2A-interacting proteins were obtained using the STRING database and shown by the STRING protein network diagram. (**B**) Gene Ontology (GO) and Kyoto Encyclopedia of Genes and Genomes (KEGG) enrichment analysis of BAZ2A and its related genes. *P* < 0.05 is considered as a statistical difference. (**C**) Analysis of the enrichment pathways of BAZ2A-related genes. (**D**) Co-expression analysis of BAZ2A and associated genes in LIHC, KIRC, KIRP, PCPG and UCEC (https://www.xiantao.love/). *P < 0.05, **P < 0.005, ***P < 0.001. *P* < 0.05 is considered as a statistical difference.

Next, we conducted a screening for BAZ2A interacting proteins and genes, followed by enrichment analyses. Through the utilization of the STRING database, The amount is 31 BAZ2A-interacting proteins were identified, and the network of interactions among these proteins is visualized in Fig. 5A. BAZ2A-related genes with the highest enrichment in biological process (BP), cellular component (CC), and molecular function (MF) are shown in Fig. 5B. The KEGG analysis revealed a markedly enrichment of BAZ2A-related genes in the lysine degradation pathway, the TGF-β signaling pathway, and viral life cycle (Fig. 5B,C). Analysis of RNAseq data from TCGA showed that BAZ2A and its related genes, KMT2D, CELF1, CCNT1, CREBBP, SMG1, and SRCAP genes, were co-expressed in LIHC, KIRC, KIRP, PCPG, and UCEC (Fig. 5D).

### BAZ2A is highly expressed in LIHC and promotes the malignant behavior of LIHC cells

**Figure 6:**
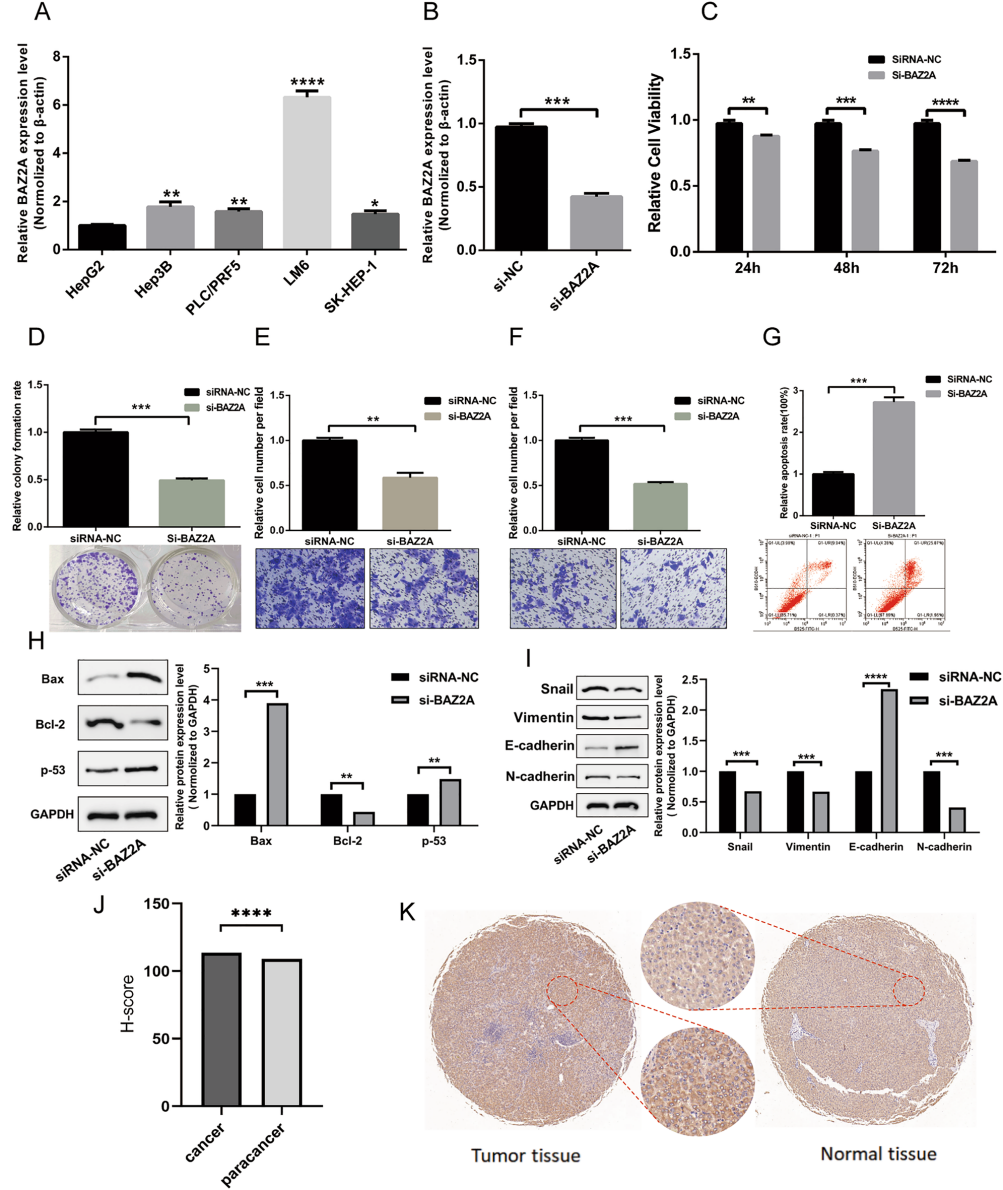
BAZ2A is upregulated in LIHC and promotes the malignant behavior of LIHC. (**A**) qRT-PCR analysis of BAZ2A mRNA in hepatoma cell lines. (**B**) Validation of siRNA effectiveness by qRT-PCR in LM6 cells. (**C**–**F**) The role of BAZ2A in the proliferation, clonal formation, migration, and invasion of LM6 cells. (**G**) Flow cytometry analysis of apoptosis in LM6 cells with BAZ2A knockdown. (**H**,**I**) Western blot analysis of apoptosis- and EMT-related molecules in LM6 cells with BAZ2A knockdown. (**J**,**K**) Immunohistochemical analysis of BAZ2A in liver cancer and paracancerous tissues. *P < 0.05, **P < 0.005, ***P < 0.001. *P* < 0.05 is considered as a statistical difference.

Through the analysis of various cancers, we found that the high expression of BAZ2A has significant effects on the survival, prognosis, immune invasion and other aspects of LIHC, while it only has significant effects on some aspects of other cancers. Therefore, we chose LIHC to further explore the role of BAZ2A in cancer. We selected LIHC to further explore the function of BAZ2A in cancer. We examined several hepatoma cell lines and found that that *BAZ2A* transcription levels were highest in LM6 cells (Fig. 6A). Thus, we selected this cell line for subsequent experiments. We designed siRNAs against *BAZ2A* and confirmed their efficacy (Fig. 6B). CCK8 and clonal formation experiments showed that *BAZ2A* downregulation inhibited cell proliferation and clonal formation of LM6 cells (Fig. 6C,D). Transwell experiments showed that *BAZ2A* silencing reduced cancer cell migration and invasion (Fig. 6E,F). Flow cytometry showed that downregulation of *BAZ2A* led to increased cell apoptosis, which was supported by western blot of Bax and other apoptosis-related proteins (Fig. 6G,H). The function of BAZ2A in facilitating cancer cell migration was further validated by the detection of epithelial–mesenchymal transition (EMT)-related molecules (Fig. 6I).

We collected tumor tissue samples from 80 patients with liver cancer and examined BAZ2A expression by immunohistochemistry. BAZ1A was highly expressed in liver cancer tissues relative to the adjacent cancer tissues in 30 samples (Fig. 6J,K).

### Multiomics analysis of BAZ2A

**Figure 7:**
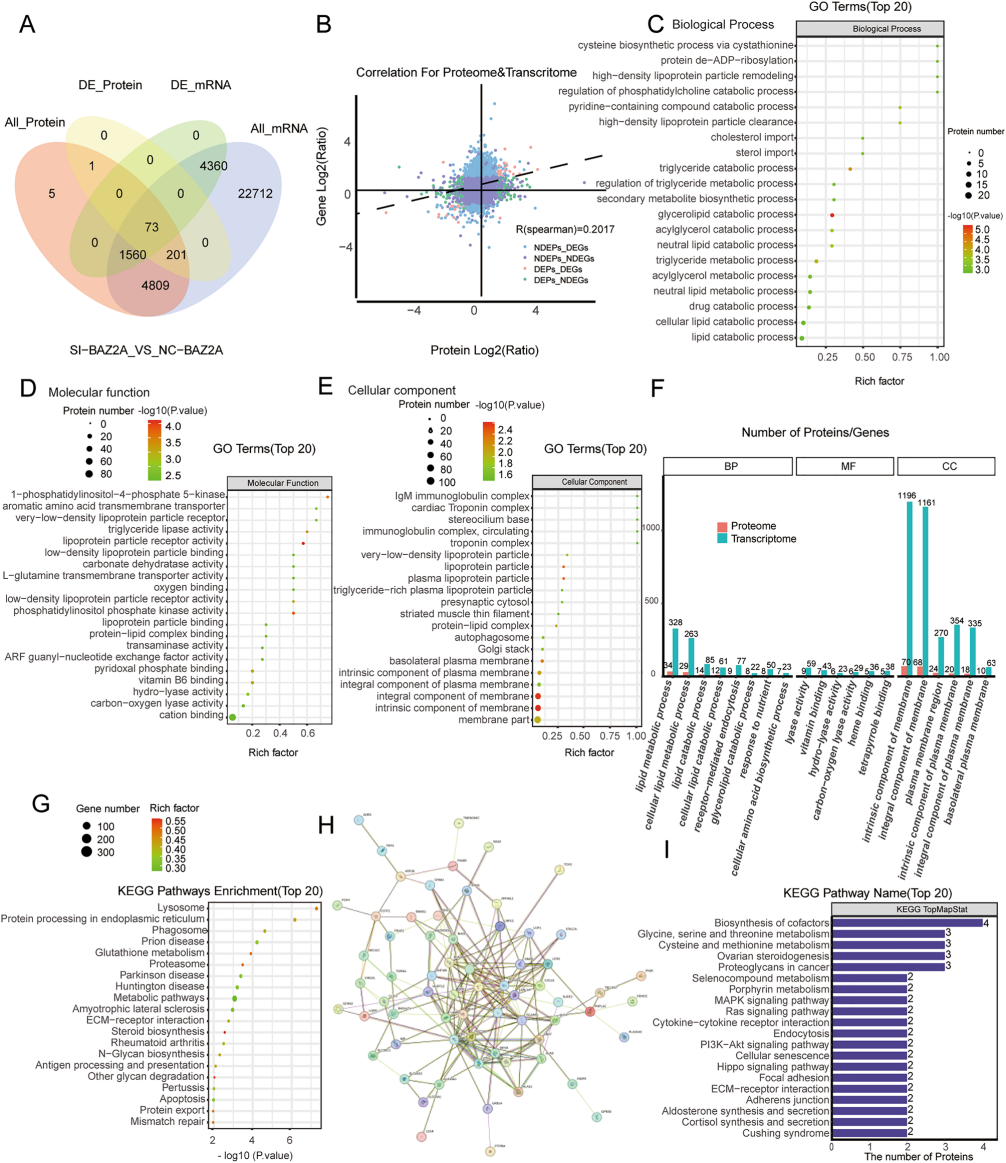
BAZ2A multiomics analysis. (**A**,**B**) Venn diagram and dot plots showing the count of DEP-DEGs identified in cancer tissue and their correlation. (**C**–**E**) GO analysis of DEPs in BP (**C**), MF (**D**), and CC (**E**). (**F**) Transcriptome analysis combined with GO enrichment analysis. (**G**) To screen for differentially expressed genes in the transcriptome, we conducted KEGG enrichment analysis. (**H**) Interactions between 73 differential proteins in (**A**) shown using STRING. (**I**) KEGG enrichment analysis of 73 DEP-DEGs. *P* < 0.05 is considered as a statistical difference.

To further validate the relationship between BAZ2A and cancer, we performed transcriptome and proteome sequencing in cells silenced for BAZ2A or transfected with NC siRNA as a negative control. The correlation analysis of transcriptomics and proteomics results indicated that upon the downregulation of BAZ2A expression, the numbers of proteins and genes with significant differences were associated, and the correlation between differential proteins and differential genes was good (Fig. 7A,B).

The differentially expressed proteins identified by proteome sequencing were subjected to analysis using GO and KEGG. GO bubble plots indicated that most of the differential proteins enriched in BP were related to lipid metabolism processes, such as cellular lipid catabolic process and triglyceride catabolic process (Fig. 7C). The differential proteins enriched in MF were related to lipoprotein receptor activity and lipoenzyme activity, such as triglyceride lipase activity and lipoprotein particle receptor activity (Fig. 7D). The differential proteins enriched in CC were associated with lipid components, including the membrane part and lipoprotein particle (Fig. 7E). These results are illustrated by directed acyclic plots in Supplementary Fig. 2A–C.

Similar results were obtained from the combined proteome and transcriptome analysis. In GO analyses with significant enrichment in both omics, BP differential genes and differential proteins were also enriched in lipid metabolism processes, with 34 proteins and 328 genes enriched in the lipid catabolic process. CC-enriched differential proteins and differential genes were associated with lipid components, with 70 proteins and 1196 genes enriched in the intrinsic component of membrane (Fig. 7F). The findings obtained were supported by transcriptome KEGG enrichment, in which the highest degree of enrichment was in the metabolic pathway (Fig. 7G). These findings support a role for BAZ2A in lipid metabolism processes.

Figure 7G shows the PPI network of the 73 differential proteins; the core protein is SOX2 (Fig. 7H). Abnormal expression of SOX2 was shown to be intricately linked to the occurrence, differentiation, metastasis, and poor prognosis of malignant tumors^19,20^. We performed KEGG enrichment analysis on the 73 differential proteins and observed that these DEPs were enriched in multiple signaling pathways of cancer, such as the RAS, MAPK signaling pathway, PI3K-AKT signaling pathway, and Hippo signaling pathway (Fig. 7I). This suggests that BAZ2A may be involved in the cancer-related processes by regulating key proteins and molecules involved in these processes.

## Discussion

Pan-cancer analysis involves the utilization of diverse databases to analyze information such as gene expression, prognosis, and mutation in different cancers; these findings are critical to provide new insights into tumor prevention and personalized treatment options^21^. Using multiple databases and tissue chip analysis, we explored the pan-cancer expression profile of BAZ2A and its potential association with prognosis and immune invasion in cancer patients. Our study suggests that BAZ2A functions as a promoter of cancer that may participate in cancer progression and promote tumor malignant behavior by regulating lipid metabolism. The results of our functional experiments in hepatoma cells further substantiate the function of *BAZ2A* in facilitating cancer cell proliferation.

Apart from alterations in BAZ2A expression observed in cancer, we also found that the *BAZ2A* gene is mutated in variety of tumor types and BAZ2A protein also exhibited changes in phosphorylation levels. Gene mutations^22,23^ and phosphorylation^24^ are common mechanisms to affect protein activity. Therefore, we speculate that the role of BAZ2A on tumorigenesis and development is not only regulated by changes in its protein levels but it may also be regulated by mutations and phosphorylation in cancer cells.

The survival analysis demonstrated that high expression of *BAZ2A* was associated with adverse prognosis in patients with tumors such as LIHC, but low expression of *BAZ2A* was associated with adverse prognosis in KIRC. This suggests that the function of *BAZ2A* in cancer may be tumor-specific, and the mechanism by which it functions in different tumors may be complex, involving different signaling pathways and related molecules. To explore its mechanism of action, we examined the transcriptome and proteome associated with BAZ2A in hepatoma cells. We also analyzed the transcriptome and metabolome of BAZ2A in other tumors, such as cervical cancer. In subsequent studies, we will further analyze the unique and common mechanisms of *BAZ2A* in different tumors.

DNA methylation is a common mechanism of regulating gene expression^25–27^. We found that in a variety of cancers, the increased expression of BAZ2A was found to be correlated with DNA methylation occurring in its promoter region.

Considering the crucial role of the tumor microenvironment on tumorigenesis and development^28–34^, the effect of *BAZ2A* on immune cell infiltration was analyzed. Our findings demonstrated that *BAZ2A* expression may be related to Treg and macrophage cell infiltration in a variety of cancers and suggested that *BAZ2A* may participate in tumor development by interacting with a variety of immune cells.

Our analysis revealed that BAZ2A exhibited elevated expression levels in tumor tissue from liver cancer patients in only 30 of 80 cases; this finding may be the small number of samples. Further investigations with a larger sample size are necessary to more comprehensively assess the expression of BAZ2A in cancer tissues and its potential correlation with various indicators.

Our results also indicated that *BAZ2A* may participate in the lipid metabolism process of cells. Thus, we speculate that *BAZ2A* may affect cell proliferation and migration through metabolic reprogramming, which may influence the occurrence and development of tumors.

## Conclusion

Our study shows that BAZ2A expression correlates with prognosis and may be a potential diagnostic marker in several cancers. BAZ2A has been implicated in facilitating tumor cell proliferation, migration, and epithelial-mesenchymal transition (EMT), while exerting inhibitory effects on apoptosis in tumor cells. Taken together, these observations suggest that BAZ2A may present as a promising therapeutic target for multiple cancer types.

## Methods

### Analysis of BAZ2A expression in tumor

*BAZ2A* gene expression was examined in data from The Cancer Genome Atlas (TCGA) using TIMER2.0^35^ (http://timer.cistrome.org/). R (version 3.6.3) and the “ggplot2” package in R were utilized for the analysis and visualization of RNAseq data. The Clinical Proteomic Tumor Analysis Consortium (CPTAC) dataset of UALCAN was employed to explore the correlation between BAZ2A expression and the pathological stage of various cancers. To investigate the variations in BAZ2A phosphorylation levels between cancerous and non-cancerous tissues, an analysis of data sourced from the UALCAN (http://ualcan.path.uab.edu/analysis-prot.html) was conducted utilizing CPTAC methods^36^.

### Survival analysis and ROC curve analysis

Kaplan–Meier^37^ was performed to evaluate the influence of *BAZ2A* expression on overall survival (OS) and disease-specific survival (DSS) (https://kmplot.com/analysis/). ROC curve analysis of RNAseq data from TCGA and GTEx was utilized to assess the significance of BAZ2A in cancer diagnosis using the R package “pROC” package and “ggplot2” to calculate the area under the curve (AUC).

### DNA methylation and mutation analysis

R language and the “ggplot2” package were used for analysis and visualization of data. Two probes of the *BAZ2A* promoter (cg12199011, cg20829193) were selected to detect its DNA methylation level.

The “Quick Search” module of the cBioPortal database was employed to select the analysis function of TCGA pan-cancer atlas, obtain the Mutation information of *BAZ2A* gene, and "Mutations" are used to obtain the specific mutation site information on the *BAZ2A* functional domain map, Click “The view of 3 D Structure” can get a 3 D model diagram (https://www.cbioportal.org/)^38^.

We used the “Comparison” module of cBioPortal to analyze the clinical outcomes of *BAZ2A* gene mutation in LIHC, KIRC and UCEC, including OS, DSS, progression-free survival (PFS), and disease-free survival (DFS).

### Immune infiltration analysis

The connection between *BAZ2A* expression and cancer-associated fibroblasts in different tumor types in TCGA database was investigated through the immune association (gene) module in the TIMER 2.0 database (http://timer.cistrome.org/).

### Enrichment analysis

The protein–protein interaction network of BAZ2A was obtained using the STRING database^39^ (http://string.embl.de/). Parameter settings were as follows: max number of interactors to show: (custom value, max interactors: 30); minimum required interaction score: [Low confidence (0.150)]; meaning of network edges: (evidence); the active interaction sources: (textmining, experiments, co-expression, neighborhood, co-occurrence). The “Similar Gene Detection” module of GEPIA 2.0 was employed for the purpose of obtaining the top 100 *BAZ2A*-related genes by comparing the differential genes between tumor and normal tissues contained in all TCGA databases. Gene Ontology (GO) and Kyoto Encyclopedia of Genes and Genomes (KEGG)^40^ data were analyzed by R packages “ggplot2” and “clusterProfiler.” Co-expression analysis of *BAZ2A, KMT2D, CELF1, CCNT1, CREBBP, SMG1,* and *SRCAP* was performed using https://portal.gdc.cancer.gov/.

### Cell culture

Hepatocellular carcinoma cells Hep-3B, HepG2, LM6, PLC/PRF/5 and SK-HEP-1 (cells verified by STR) were all provided by Saier Biolabs (Tianjin, China). The cells were subjected to culturing in an incubator set at 37 °C with 5% CO_2_ using RPMI-1640 medium (Thermo, Waltham, MA, USA) supplemented with 10% FBS (Thermo). SiRNAs (si-NC and si-BAZ2A) procured from Saier Biotechnology Inc (Tianjin, China) were transfected into cells using Lipofectamine 2000 (Thermo). Following a 4–6 h incubation in medium without serum, the cells were subsequently switched to medium supplemented with serum. The sequences of siRNAs: si-BAZ2A: 5′-GAGAGUGUCAGACUACUAUTT-3′ and si-NC: 5′-UUCUCCGAACGUGUCACGUTT-3′.

### RT-qPCR

The cells were subjected to RNA extraction using Trizol (Thermo) reagent. To synthesize cDNA from RNA, the FastKing RT kit (Takara, Chuo-ku, Osaka City, Japan) was employed. The SYBR Premix EX Taq Kit was utilized for RT-PCR analysis (Takara). *BAZ2A* mRNA was quantified by the 2^−ΔΔCT^ method, with β-actin mRNA as an internal reference. The reaction was carried out under the following conditions: an initial denaturation at 94 °C for 30 s, followed by annealing at 58 °C for 30 s and extension at 72 °C for 30 s. The procedure was repeated for a total of 40 times.

Below are the primer sequences that were used: β-actin, sense (5′-CGTGACATTAAGGAGAAGCTG-3′) antisense (5′-CTAGAAGCATTTGCGGTGGAC-3′); BAZ2A, sense (5′-GGAGCAGCGGGTTATCAT-3′) and antisense (5′-CACAGCCAGGTCCAAAGG-3′).

### CCK8

After being transfected with siRNA, inoculated into 96-well plates at a concentration of 2 × 10^3^ cells per well, followed by incubation for 24 h. PBS was used to perform a wash on the cells. The cells were treated with CCK8 reagent (Thermo) and incubated for a period of 2 h. The OD value was determined on a microreader.

### Western blot

After lysing the cells, the protein concentration was assessed utilizing the BCA Quantitation Kit (Thermo). Subsequently, an equal amount of protein was resolved by 6% SDS-PAGE and transferred onto a nitrocellulose filter membrane. At room temperature, the membrane was treated in 3% skim milk for 2 h to block non-specific binding. In this study, due to multiple antibodies incubated at the same time will interfere with each other, resulting in mixed bands, the membrane is cut and underwent overnight incubation at 4 °C with specific primary antibodies respectively: BAZ2A (ab290639, Abcam, Cambridge, MA, USA), GADPH (ab8245, Abcam), BAX (ab32503,Abcam), Snail (MA5-14801, Thermo), p53 (GTX34938, GeneTex, TX, USA), Bcl-2 (GTX100064, GeneTex), Vimentin (GTX40346,GeneTex), E-cadherin (CSB-RA576116A0HU, CUSABIO, Wuhan, China), and N-cadherin (CSB-RA243509A0HU, CUSABIO). The membrane was subjected to 1 h of incubation with the corresponding secondary antibody (CSB-PA564648/CSB-PA573747, CUSABIO). ECL chemiluminescence substrate (PerkinElmer, Waltham, MA, USA) was used to detect bands.

### Transwell assays

1 × 10^5^ cells suspended in medium without serum at a concentration of 100 µL were added to the upper chamber of a Transwell apparatus, and 750 µL serum-containing medium was introduced into the lower chamber. Three biological replicates for each condition were included. Plates were incubated for 12–16 h and the chambers were removed. The filter was fixed with 4% paraformaldehyde (BL539A, Biosharp, Beijing, China) and incubated with 800 µL of 0.5% crystal violet (G1063, Solarbio, Beijing, China) solution for 15 min under dark conditions. Samples were observed using an inverted microscope. Each sample was randomly examined in five different fields of view, and the numbers of cells passing through the filtration membrane were counted. For invasion assays, Matrigel (Corning, NY, USA) diluted 1:8 with serum-free medium was introduced into the lower chamber and incubated for 5 h in a 37 °C incubator. The cells were then evaluated as described above.

### Clonal formation assays

Cells were seeded into six-well plates (50, 100, or 200 cells per well) and cultured for 2–3 weeks. After removal of the medium, the plates were rinsed twice with PBS, followed by fixation in 5 mL of pure methanol for 15 min. After removing the fixative solution, the cells were subjected to treatment with 0.4% crystal violet stain for a duration of 10–30 min. Plates were washed and air-dried, and photographs were taken. The count of colonies was performed to determine their quantity.

### Flow cytometry

Cells were harvested and then resuspended in 195 µL of Annexin V-FITC solution (Beyotime, Shanghai, China), followed by gentle mixing with 5 µL Annexin V-FITC. The sample was incubated with propidium iodide staining solution and incubated for 10–20 min on ice protected from light. Cells were evaluated on a flow cytometer (FACS Calibur, BD BioSciences).

### Immunohistochemistry

Paraffin sections were heated overnight at 60 °C after xylene dewaxing, ethanol dehydration, and PBS washing. Tissue sections were boiled in boiling buffer solution (pH 6.0) for antigen retrieval. Tissue samples were blocked using PBS containing 5–10% normal sheep serum at room temperature and then incubated in primary antibody working solution at 4 °C overnight. Following PBS washing, the samples were exposed to secondary antibodies and incubated for 30 min. After another round of PBS washing, 3,3′ diaminobenzidine tetrahydrochloride was used for staining, and hematoxylin was subsequently applied as a counterstain. The stained samples were then examined under a microscope.

This study enrolled a cohort of 80 patients who had been diagnosed with HCC. Prior to their participation, all patients provided informed consent prior to participation. The Ethics Committee of North China University of Science and Technology/Tangshan People's Hospital/Zunhua People’s Hospital granted approval of the research protocol.

### Transcriptome sequencing analysis

The collection of samples was conducted using Trizol Reagent (Invitrogen, Carlsbad, CA USA) at a 5 × 10^6^ cells/mL. Transcriptome sequencing was performed by Zhongke New Life Biotechnology Co., Ltd (China).

### Tandem mass tag (TMT)-labeled quantitative proteomics

Cells were lysed using RIPA lysis buffer (Thermo). Next 1–10 × 10^7^ cells were loaded into precooled centrifuge tubes for 4D label-free quantitative proteomics.

The proteome sequencing was performed by Zhongke New life Biotechnology Co., Ltd.

### Statistical analysis

The statistical analysis was performed using GraphPad Prism 8.2.1, and the comparison between the groups was performed using Student’s *t*-test. *P* < 0.05 was statistically significant.

### Ethical conduct of research

The study follows the principles of the Declaration of Helsinki. Liver cancer samples and clinical data were collected from patients undergoing surgery at Tangshan People’s Hospital and Zunhua People's Hospital, and informed consent was obtained from all patients included in this study. All studies performed with human tissue specimens were approved by the Ethics Committee of North China University of Science and Technology.

### Supplementary Information

Supplementary Information.

Supplementary Figures.

## Data Availability

The datasets generated during and/or analyzed during the current study are available from the corresponding author on reasonable request.
