# Supplementary figures and images for: Pan-cancer and multi-omics analyses revealed the diagnostic and prognostic value of BAZ2A in liver cancer
Source: Sci Rep. 2024 Mar 4;14:5228. doi: 10.1038/s41598-024-56073-7 (PMC10909891; doi:10.1038/s41598-024-56073-7)

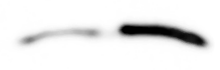

Supplement: Supplementary file 1 — Supplementary Information. [file 41598_2024_56073_MOESM1_ESM.zip › Apoptosis Crop drawing/Bax-裁剪图.tiff]

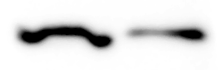

Supplement: Supplementary file 1 — Supplementary Information. [file 41598_2024_56073_MOESM1_ESM.zip › Apoptosis Crop drawing/Bcl-2-裁剪图.tiff]

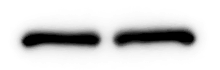

Supplement: Supplementary file 1 — Supplementary Information. [file 41598_2024_56073_MOESM1_ESM.zip › Apoptosis Crop drawing/GAPDH-裁剪图.tiff]

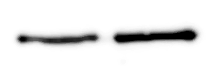

Supplement: Supplementary file 1 — Supplementary Information. [file 41598_2024_56073_MOESM1_ESM.zip › Apoptosis Crop drawing/P-53-裁剪图.tiff]

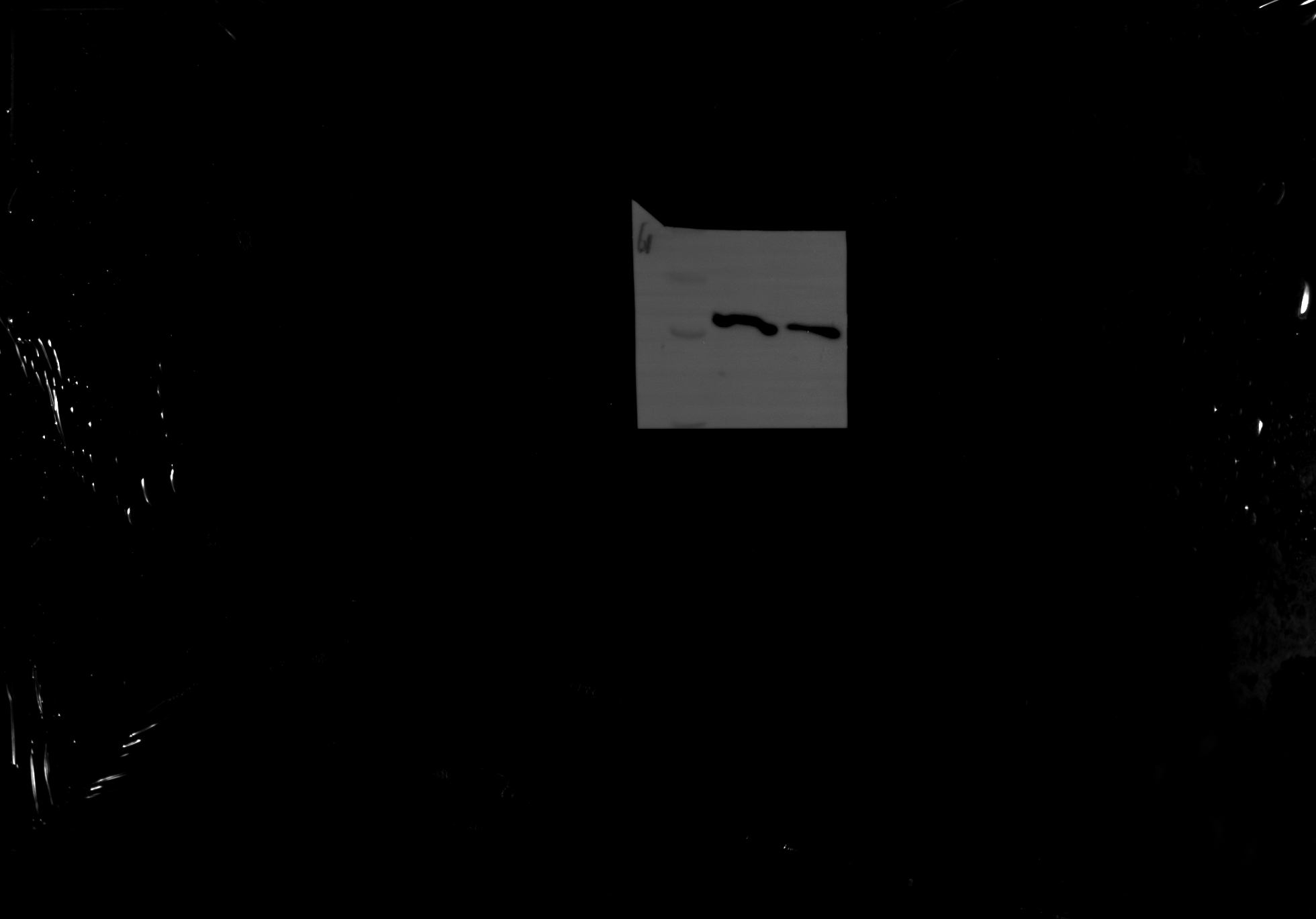

Supplement: Supplementary file 1 — Supplementary Information. [file 41598_2024_56073_MOESM1_ESM.zip › Apoptosis/Bcl-2-merge.tif]

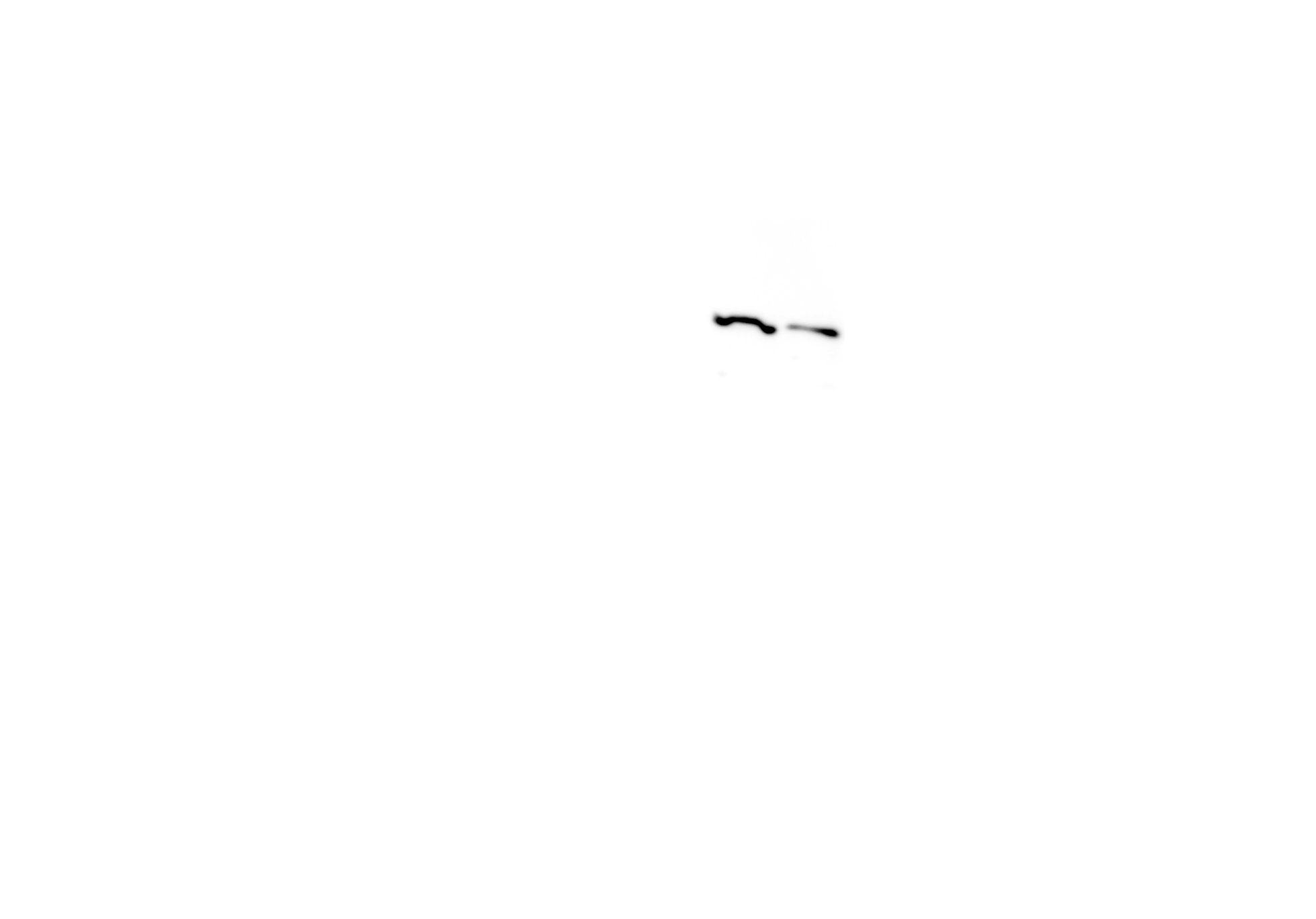

Supplement: Supplementary file 1 — Supplementary Information. [file 41598_2024_56073_MOESM1_ESM.zip › Apoptosis/Bcl-2-sample.tif]

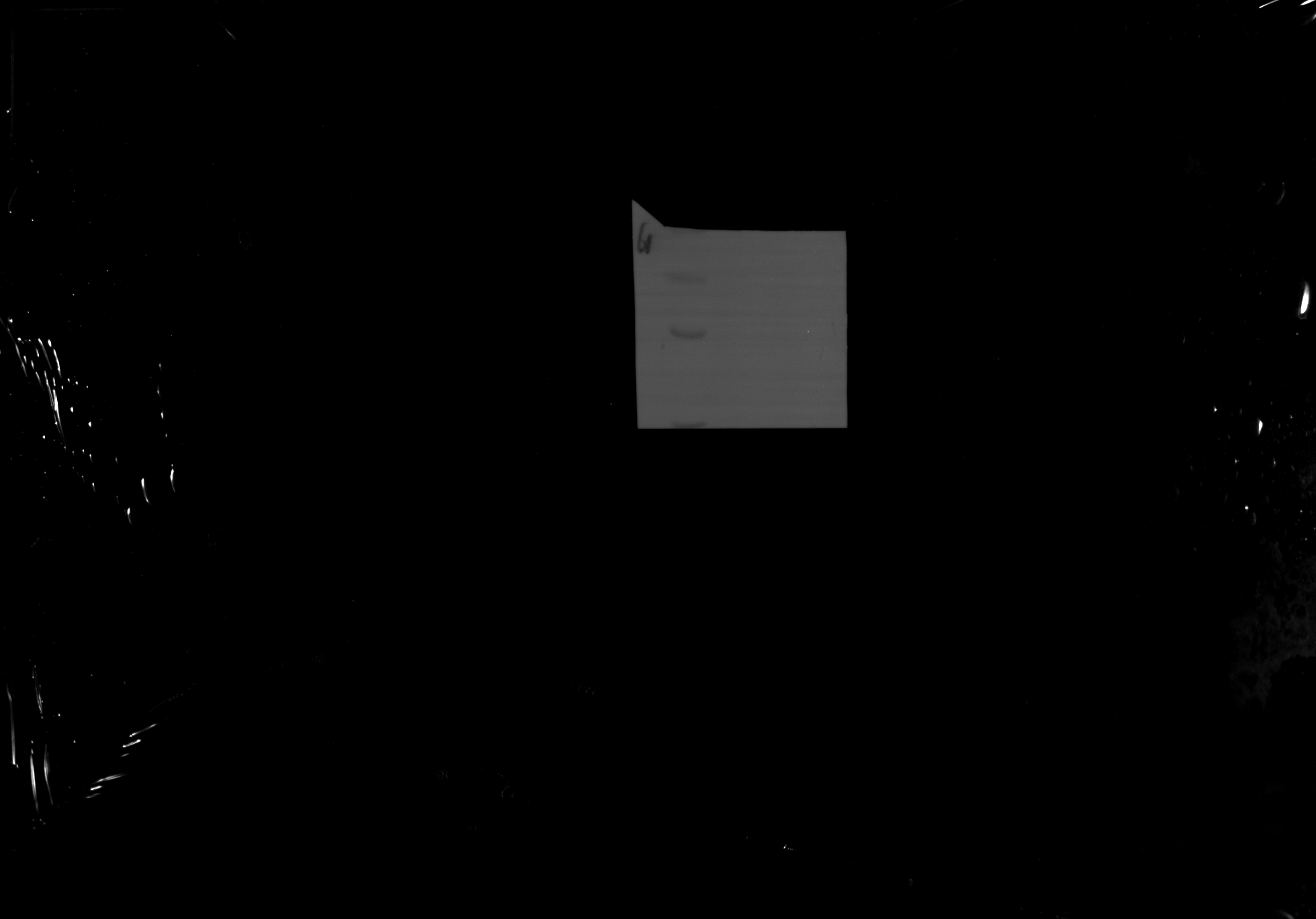

Supplement: Supplementary file 1 — Supplementary Information. [file 41598_2024_56073_MOESM1_ESM.zip › Apoptosis/Bcl-2-white.tif]

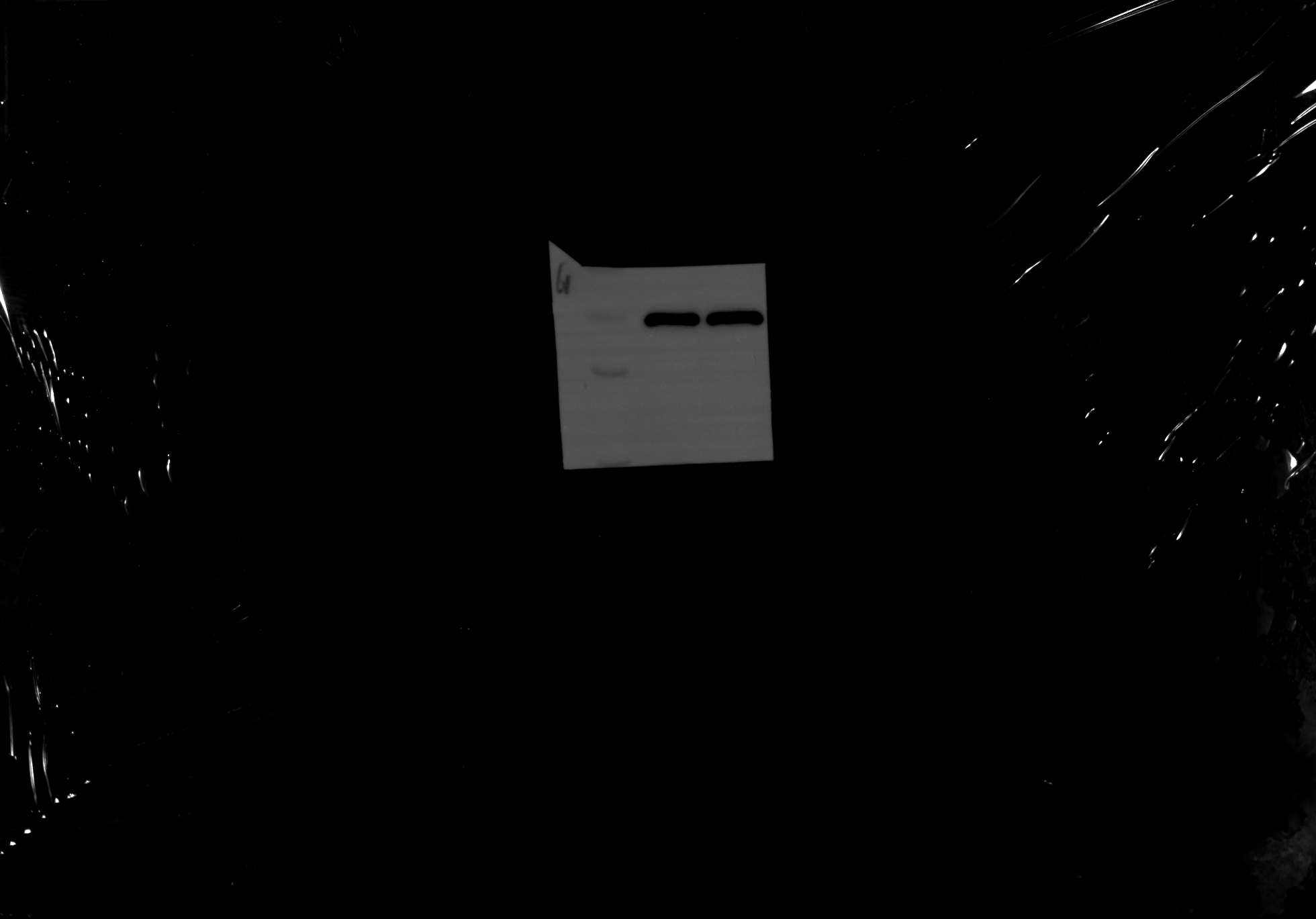

Supplement: Supplementary file 1 — Supplementary Information. [file 41598_2024_56073_MOESM1_ESM.zip › Apoptosis/GAPDH-merge.tif]

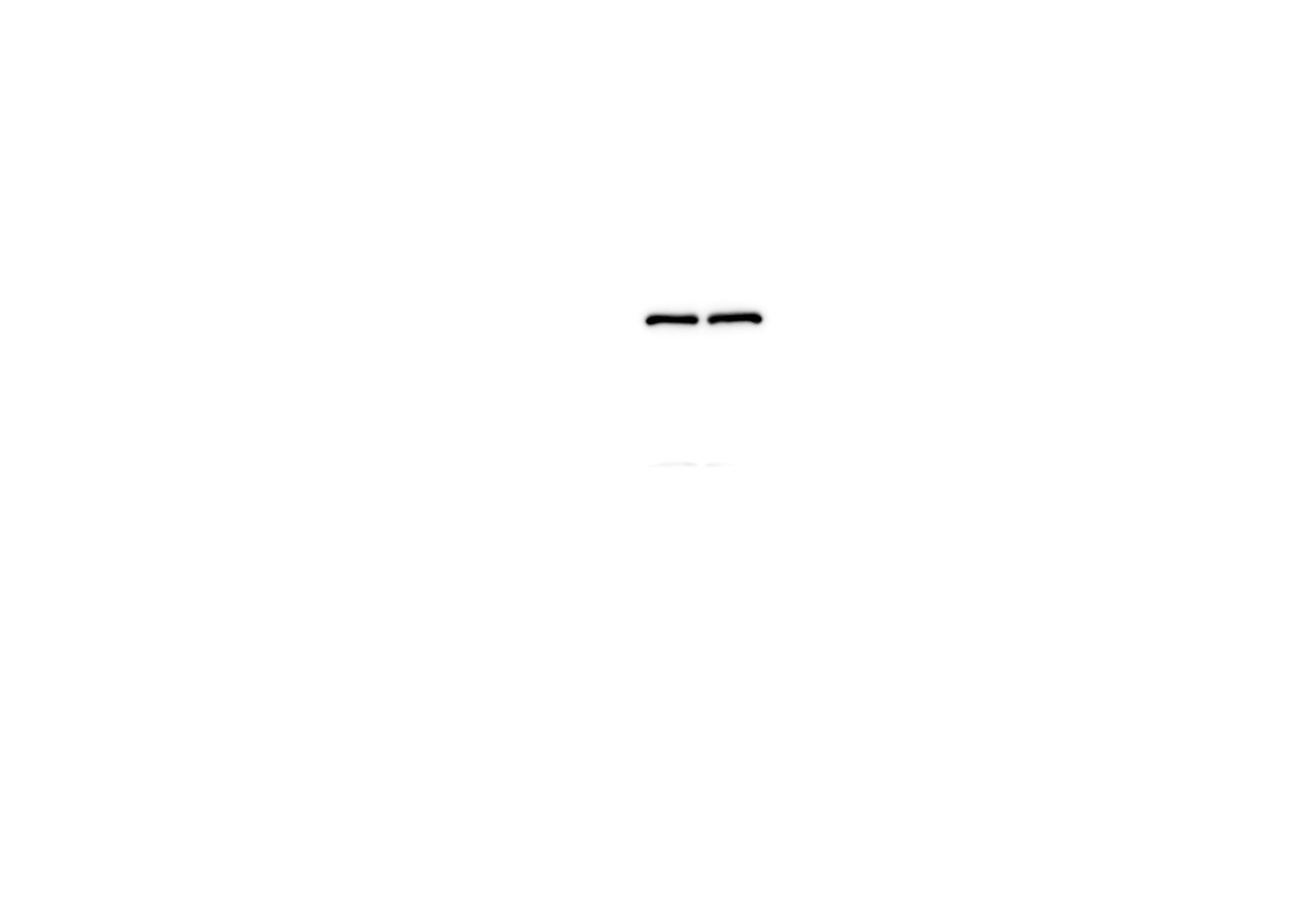

Supplement: Supplementary file 1 — Supplementary Information. [file 41598_2024_56073_MOESM1_ESM.zip › Apoptosis/GAPDH-sample.tif]

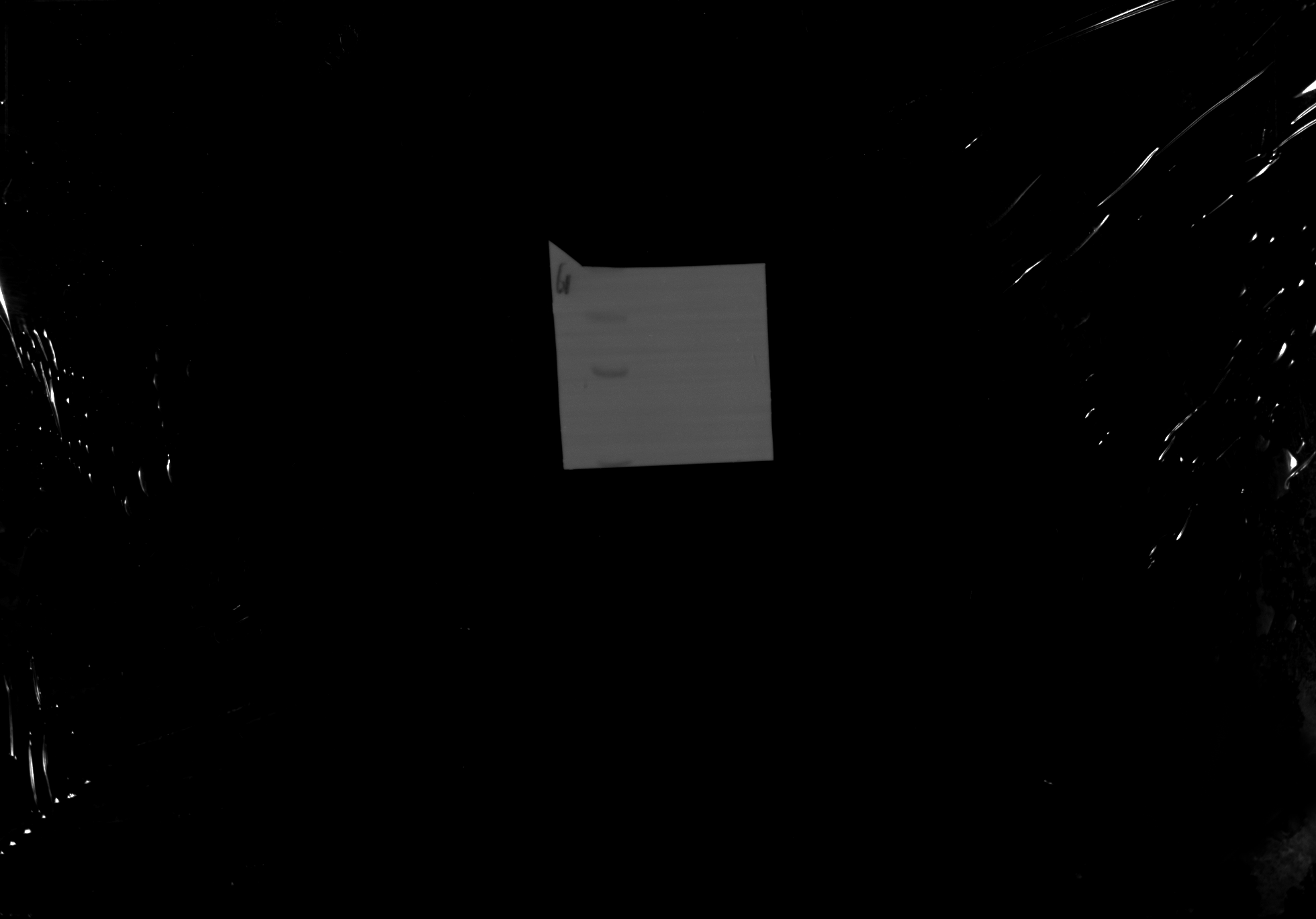

Supplement: Supplementary file 1 — Supplementary Information. [file 41598_2024_56073_MOESM1_ESM.zip › Apoptosis/GAPDH-white.tif]

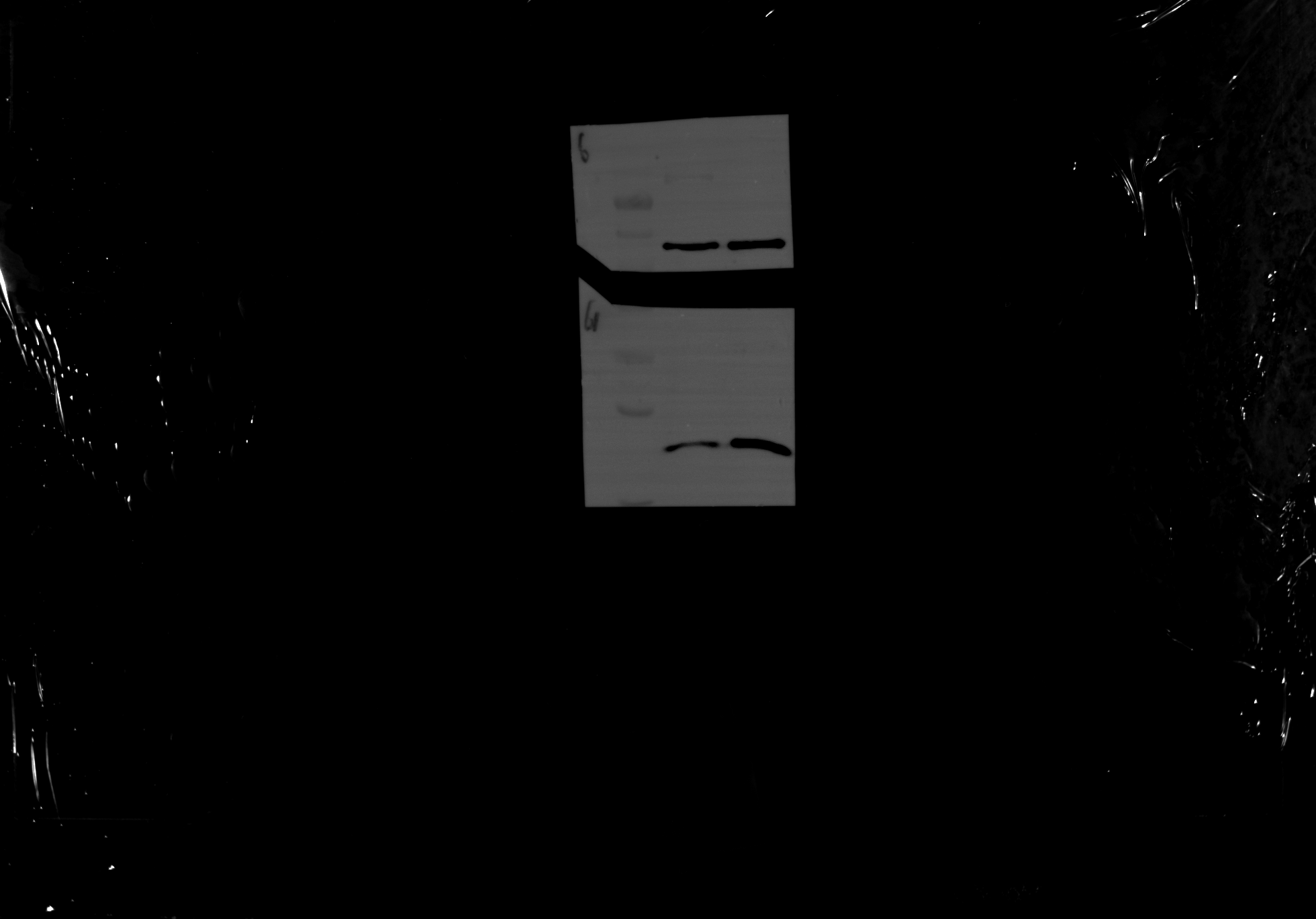

Supplement: Supplementary file 1 — Supplementary Information. [file 41598_2024_56073_MOESM1_ESM.zip › Apoptosis/P-53-Bax-Merge.tif]

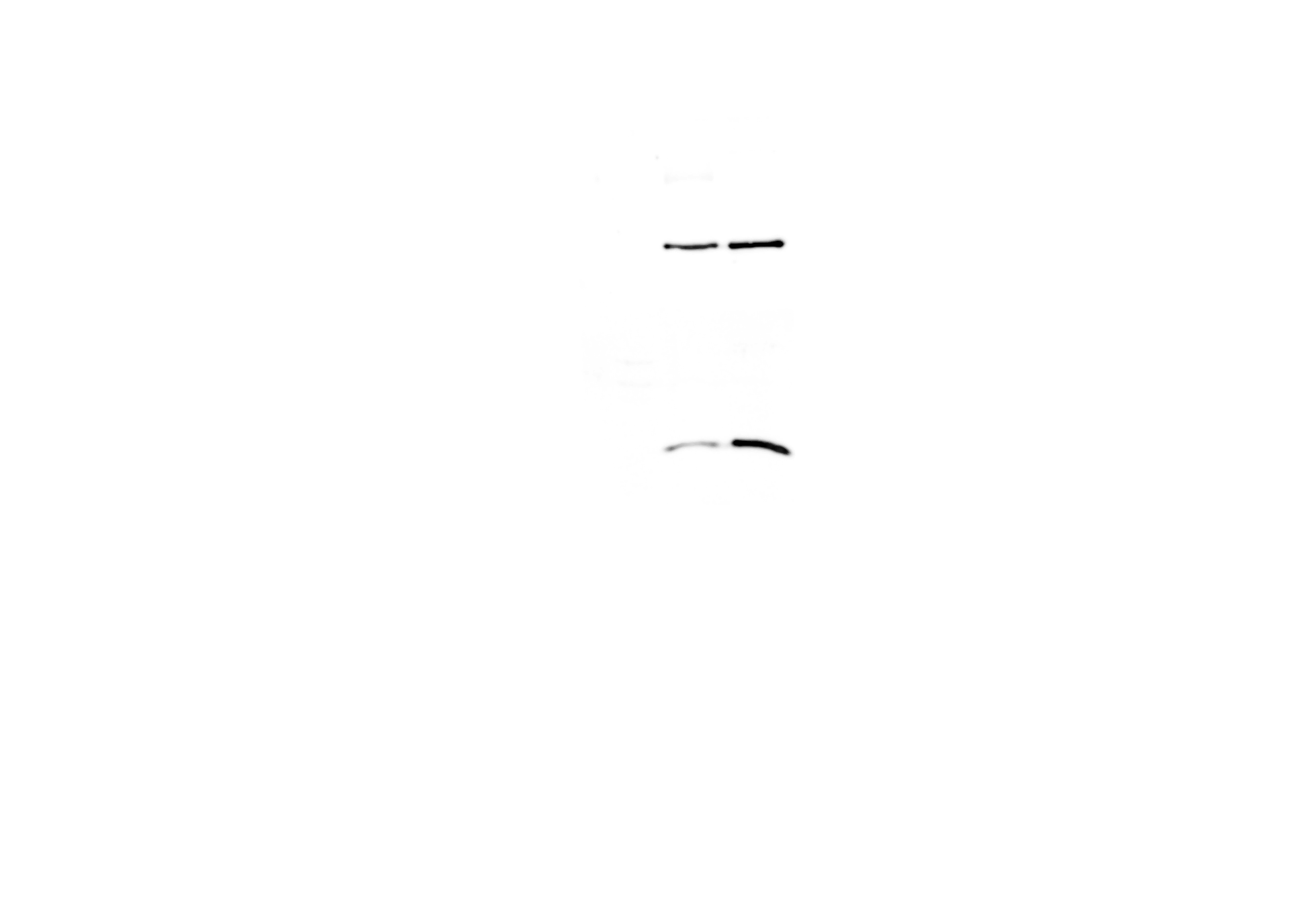

Supplement: Supplementary file 1 — Supplementary Information. [file 41598_2024_56073_MOESM1_ESM.zip › Apoptosis/P-53-Bax-sample.tif]

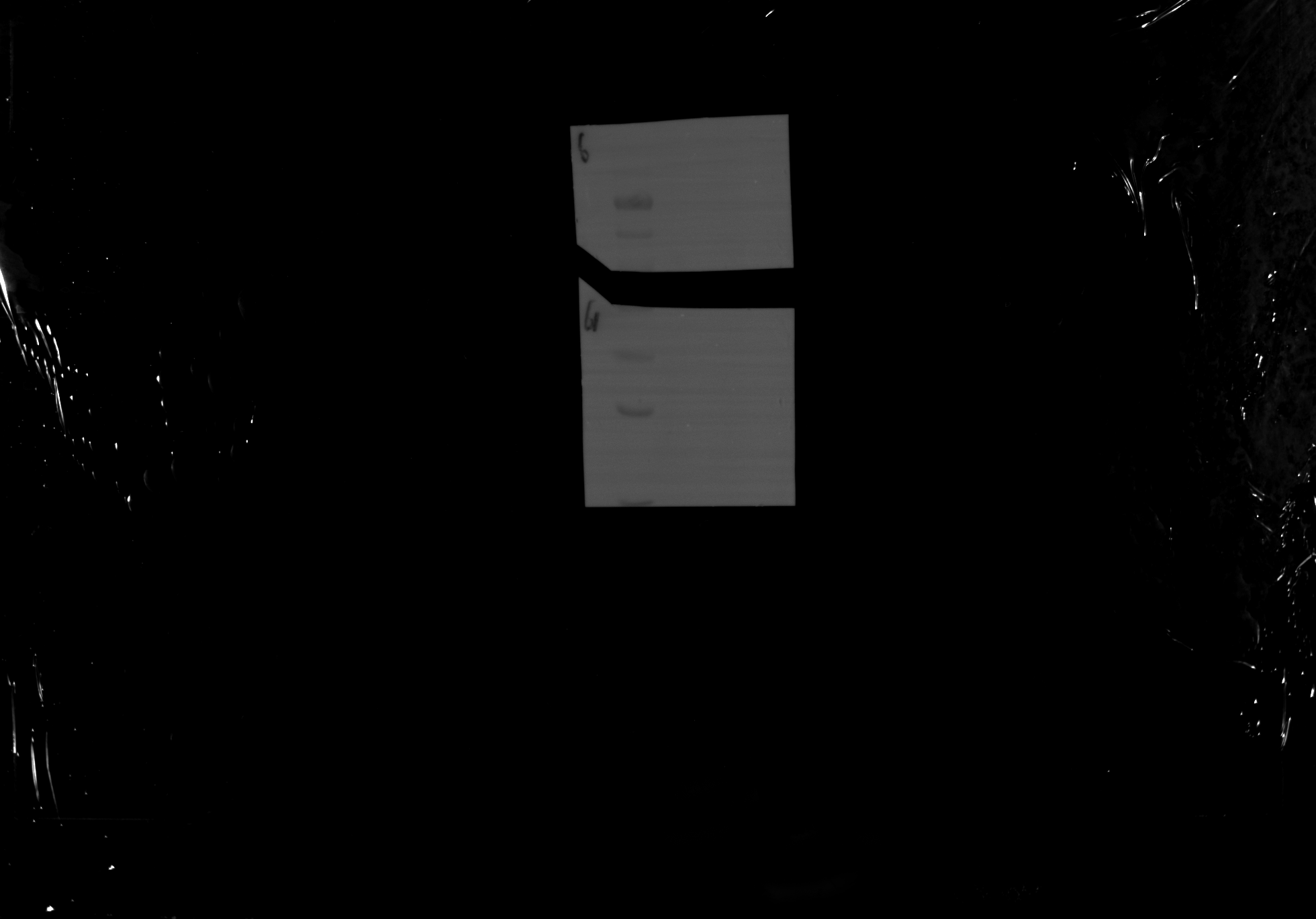

Supplement: Supplementary file 1 — Supplementary Information. [file 41598_2024_56073_MOESM1_ESM.zip › Apoptosis/P-53-Bax-white.tif]

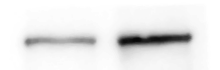

Supplement: Supplementary file 1 — Supplementary Information. [file 41598_2024_56073_MOESM1_ESM.zip › EMT Crop drawing/E-cad-裁剪图.tiff]

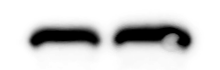

Supplement: Supplementary file 1 — Supplementary Information. [file 41598_2024_56073_MOESM1_ESM.zip › EMT Crop drawing/GAPDH-裁剪图.tiff]

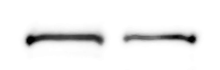

Supplement: Supplementary file 1 — Supplementary Information. [file 41598_2024_56073_MOESM1_ESM.zip › EMT Crop drawing/N-cad-裁剪图.tiff]

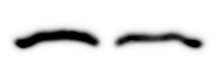

Supplement: Supplementary file 1 — Supplementary Information. [file 41598_2024_56073_MOESM1_ESM.zip › EMT Crop drawing/Snail-裁剪图.tiff]

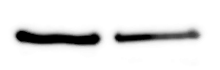

Supplement: Supplementary file 1 — Supplementary Information. [file 41598_2024_56073_MOESM1_ESM.zip › EMT Crop drawing/Vim-裁剪图.tiff]

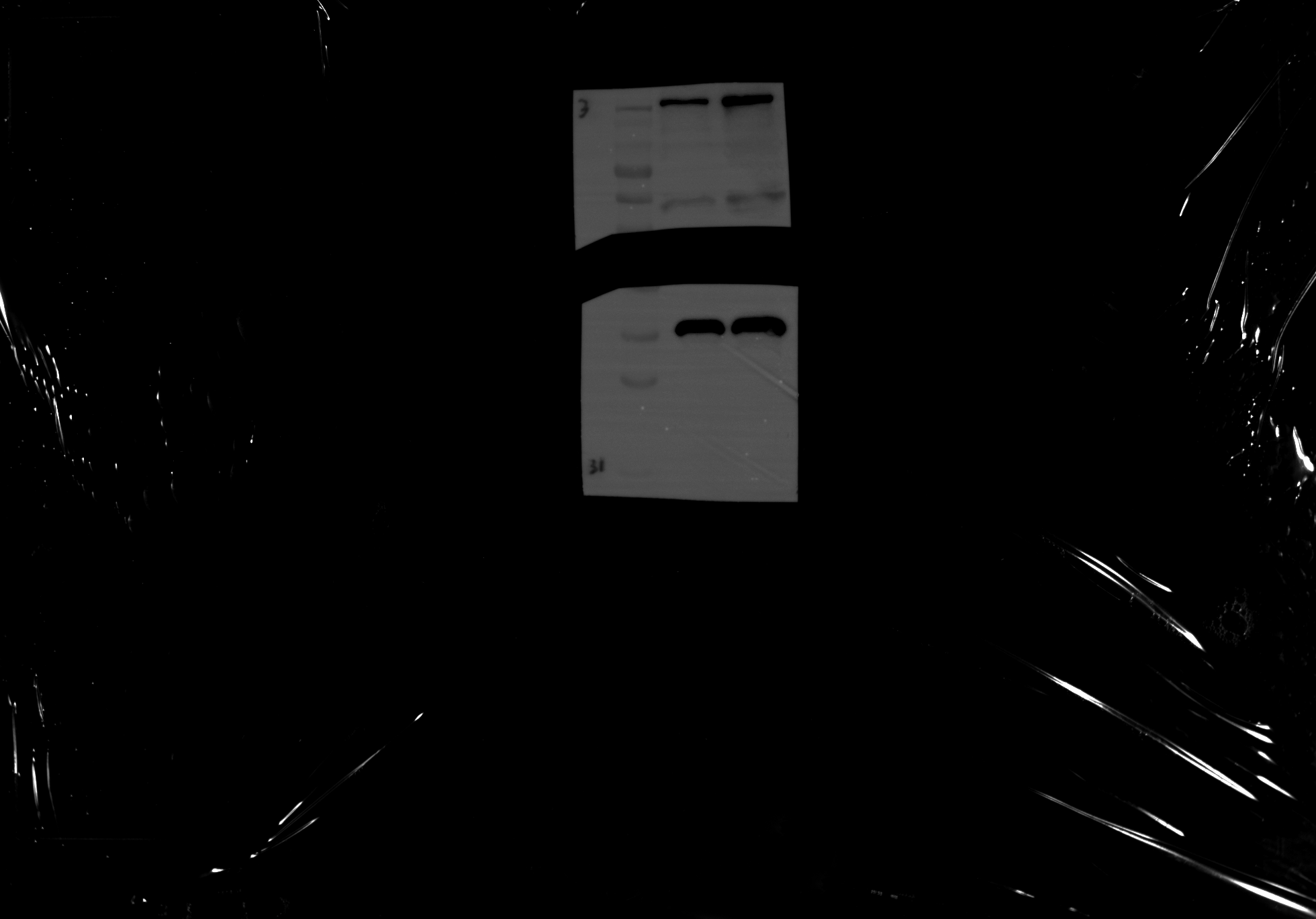

Supplement: Supplementary file 1 — Supplementary Information. [file 41598_2024_56073_MOESM1_ESM.zip › EMT√/E-cad-GAPDH-Merge.tif]

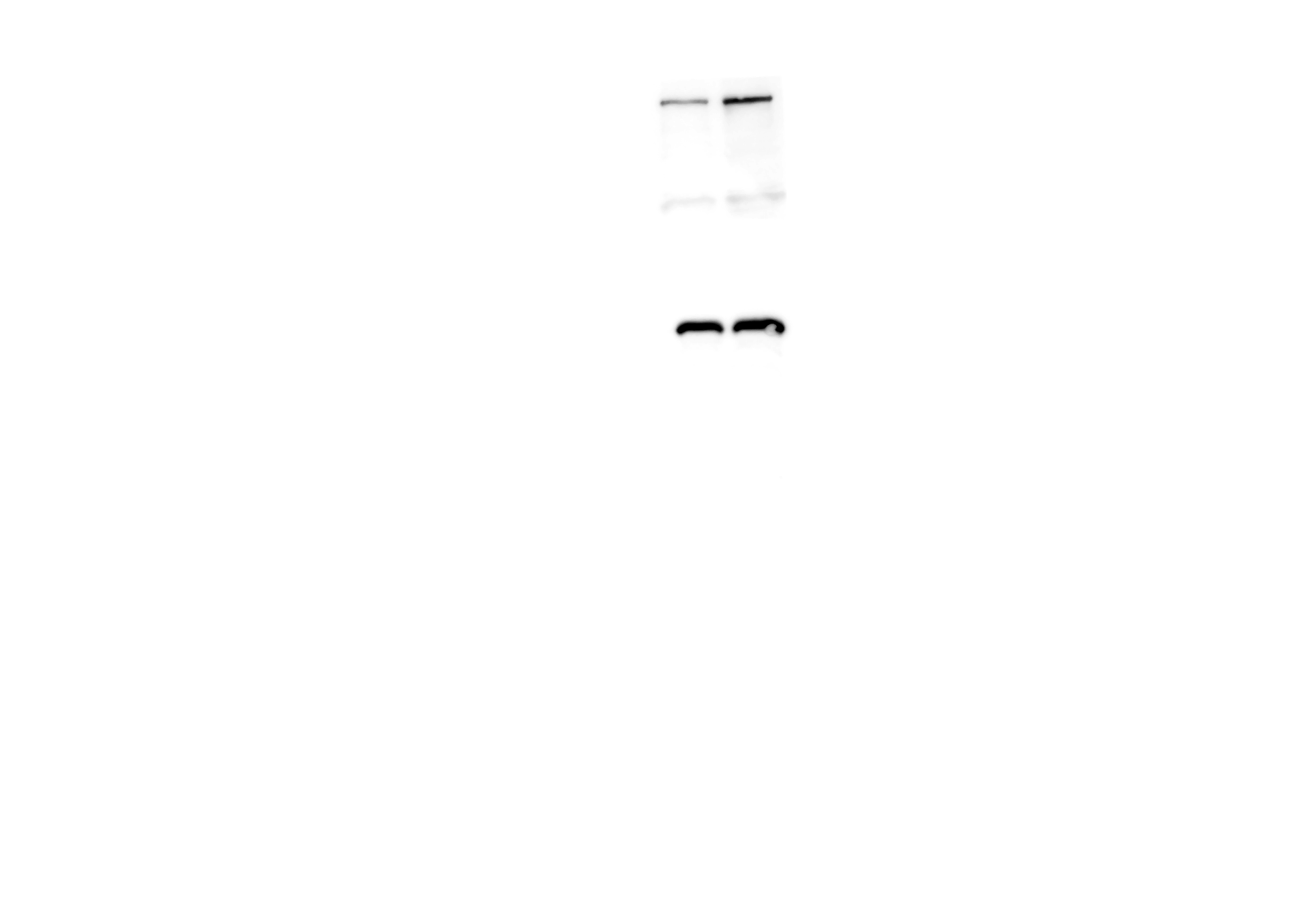

Supplement: Supplementary file 1 — Supplementary Information. [file 41598_2024_56073_MOESM1_ESM.zip › EMT√/E-cad-GAPDH-sample .tif]

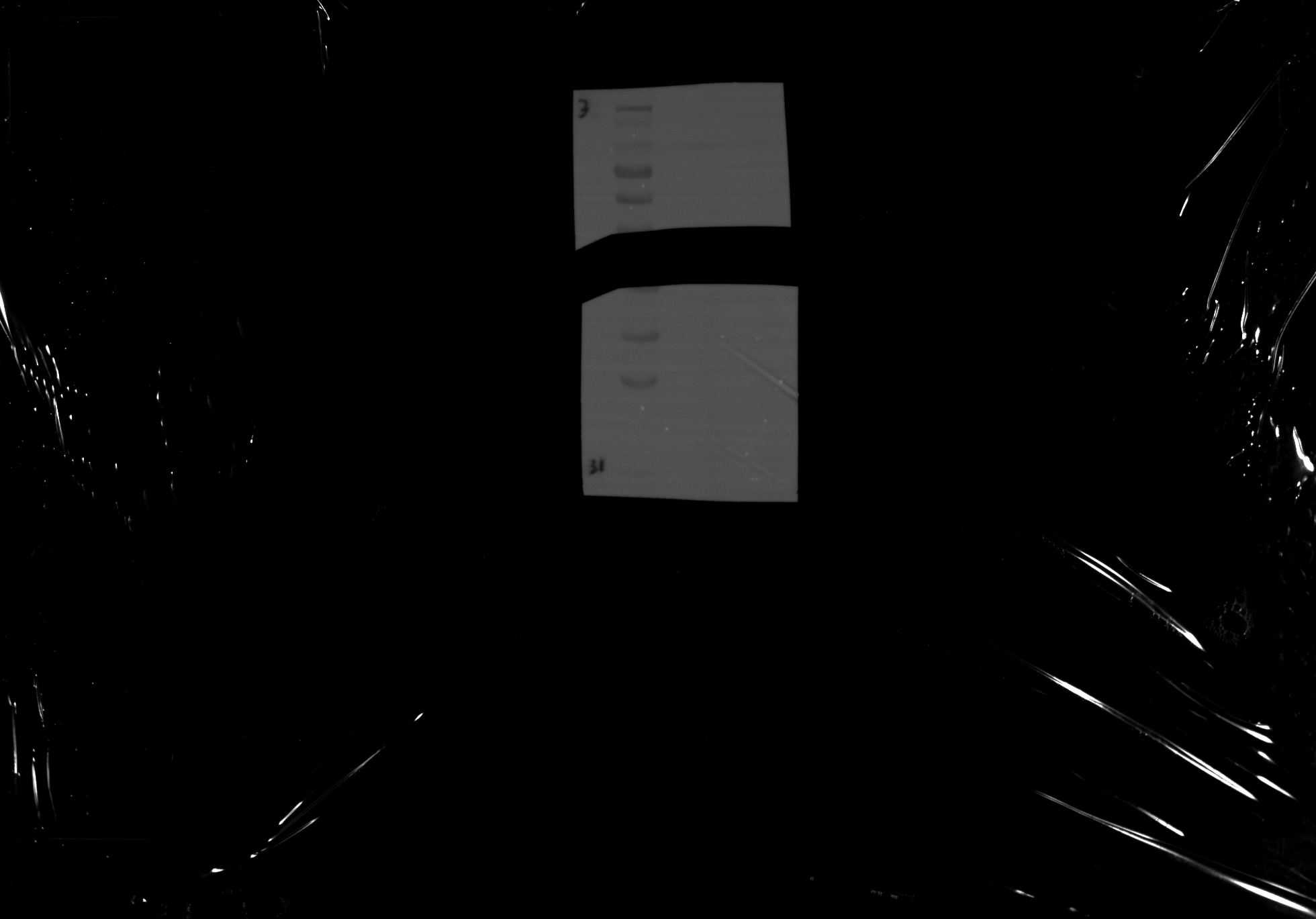

Supplement: Supplementary file 1 — Supplementary Information. [file 41598_2024_56073_MOESM1_ESM.zip › EMT√/E-cad-GAPDH-white.tif]

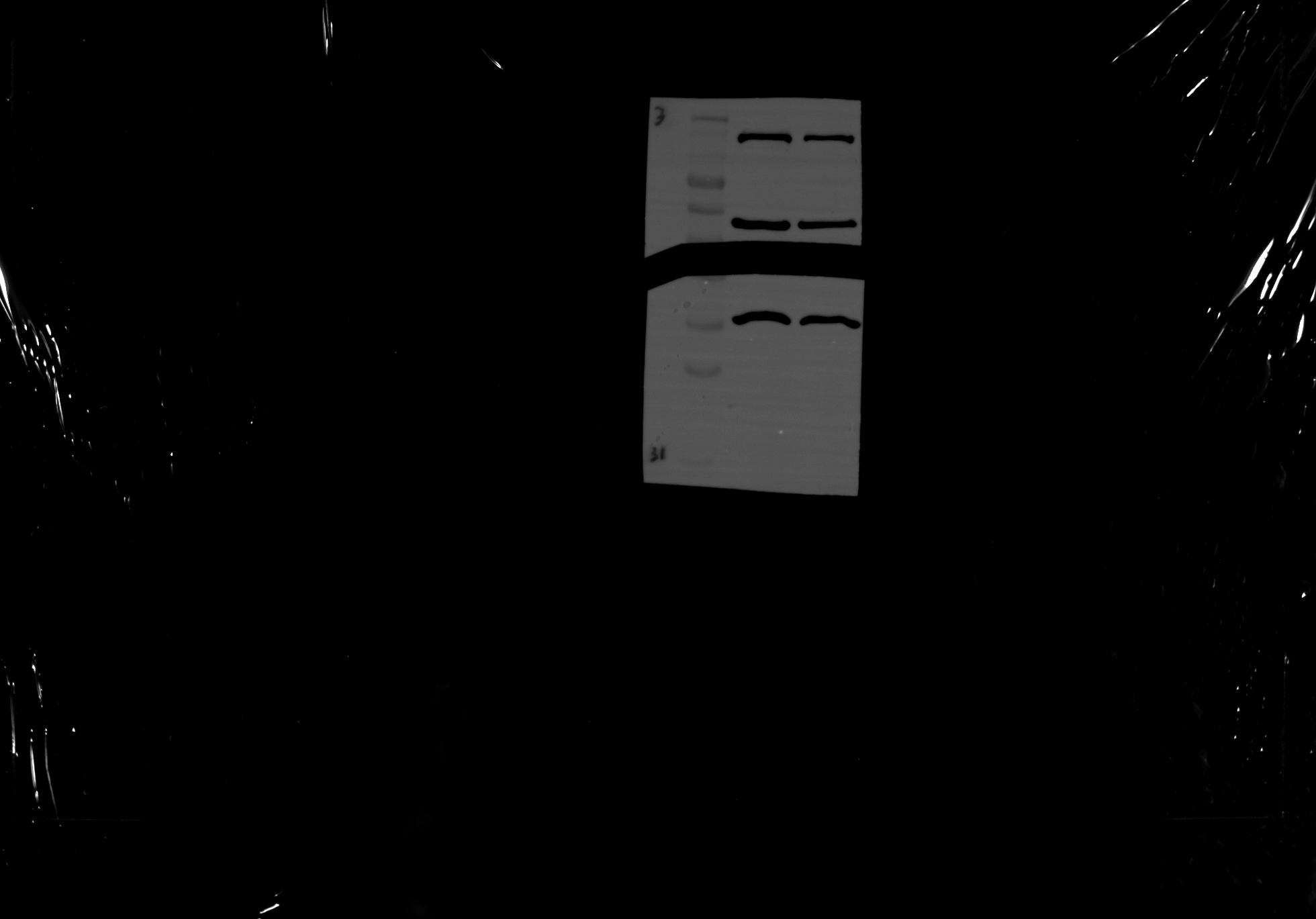

Supplement: Supplementary file 1 — Supplementary Information. [file 41598_2024_56073_MOESM1_ESM.zip › EMT√/N-cad-vim-Snail-Merge.tif]

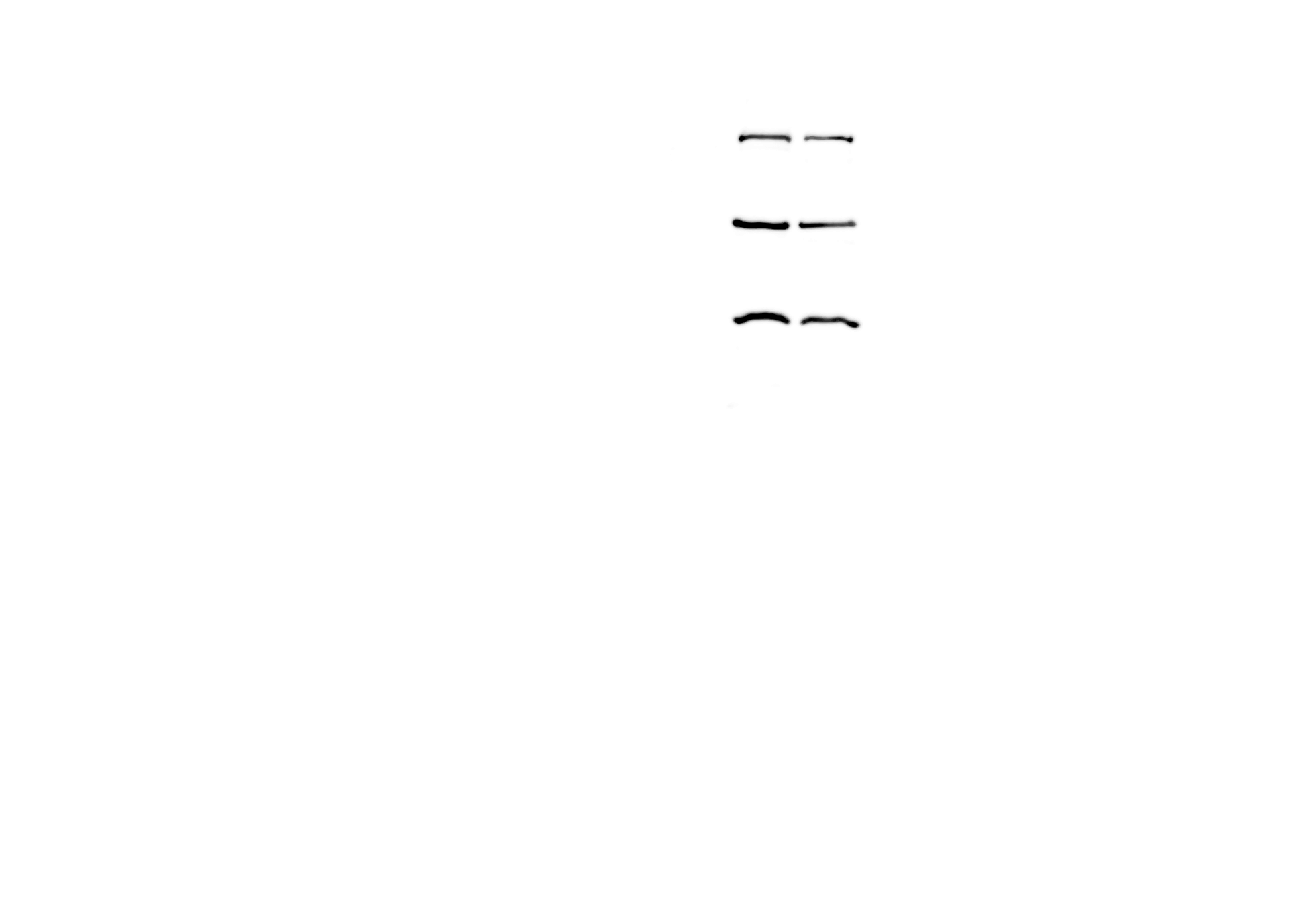

Supplement: Supplementary file 1 — Supplementary Information. [file 41598_2024_56073_MOESM1_ESM.zip › EMT√/N-cad-vim-Snail-sample.tif]

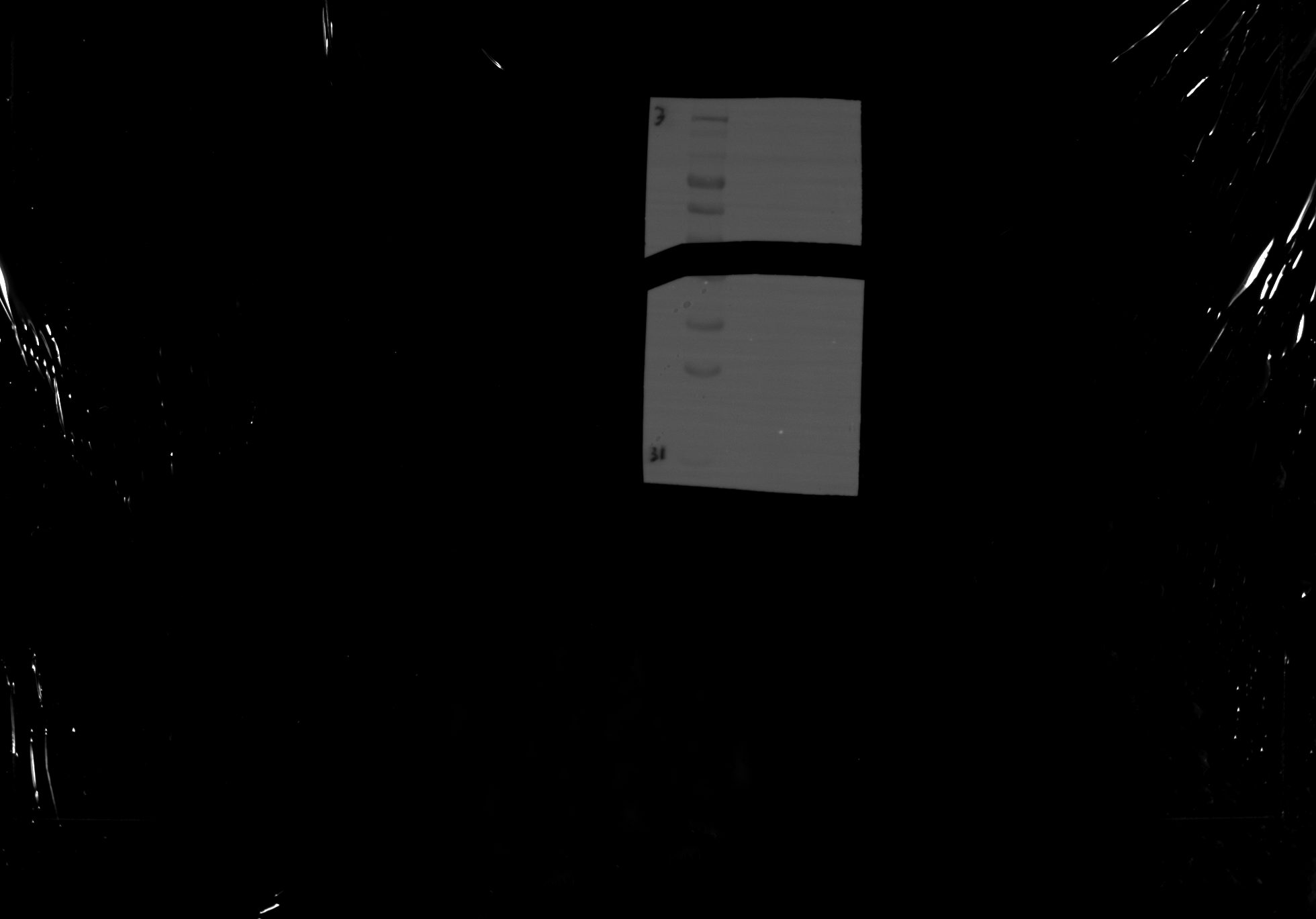

Supplement: Supplementary file 1 — Supplementary Information. [file 41598_2024_56073_MOESM1_ESM.zip › EMT√/N-cad-vim-Snail-white.tif]
