# Supplementary material for: Pan-cancer and multi-omics analyses revealed the diagnostic and prognostic value of BAZ2A in liver cancer
Source: Sci Rep. 2024 Mar 4;14:5228. doi: 10.1038/s41598-024-56073-7 (PMC10909891; doi:10.1038/s41598-024-56073-7)

**Supplementary Figure 1.**

The correlation between BAZ2A protein expression and pathological stage and differences in BAZ2A protein phosphorylation between tumor and non-tumor (normal) groups. (A) The relationship between BAZ2A protein expression and pathological stage was analyzed in clear cell carcinoma, UCEC, ovarian cancer, and colon cancer. *: $p < 0.05$; **: $p < 0.01$; ***: $p < 0.001$. (B) BAZ2A phosphorylation levels differ between tumor and the corresponding adjacent normal tissues in LIHC, KIRC, OV and HNSC. Results were retrieved from the UALCAN database. *: $p < 0.05$; **: $p < 0.01$; ***: $p < 0.001$.

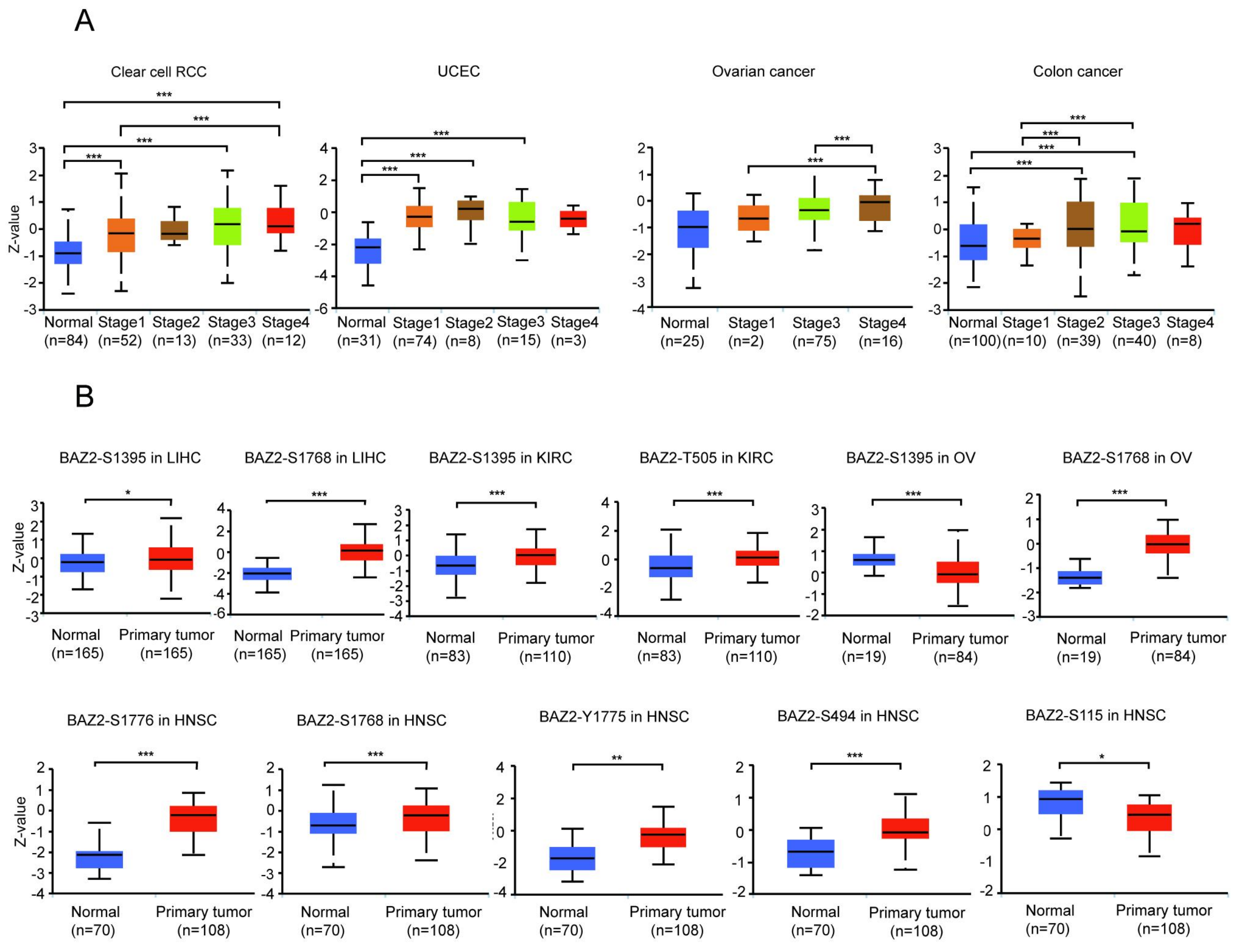

**Supplementary Figure 2.**

BAZ2A multiomics analysis. (A–C) The directed acyclic plots illustrate the GO analysis of DEPs in BP (A), MF (B), and CC (C).

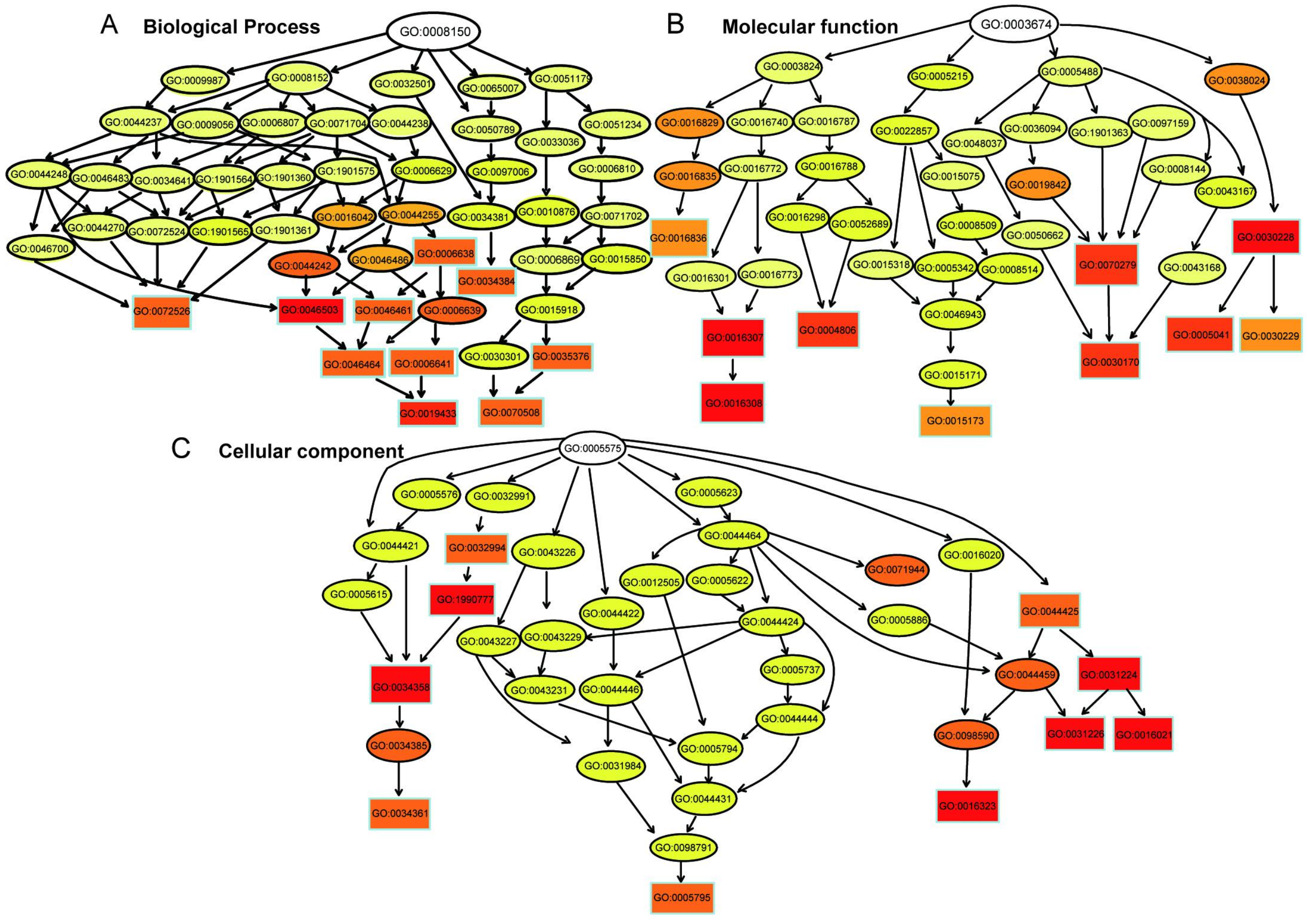

Supplement: Supplementary file 2 — Supplementary Figures. [file 41598_2024_56073_MOESM2_ESM.pdf]
